# Engineered extracellular vesicles derived from pluripotent stem cells: a cell-free approach to regenerative medicine

**DOI:** 10.1093/burnst/tkaf013

**Published:** 2025-02-11

**Authors:** Aline Yen Ling Wang, Huang-Kai Kao, Yen-Yu Liu, Charles Yuen Yung Loh

**Affiliations:** Center for Vascularized Composite Allotransplantation, Chang Gung Memorial Hospital, No. 5, Fuxing St., Guishan District, Taoyuan City 333, Taiwan, China; Department of Plastic and Reconstructive Surgery, Chang Gung Memorial Hospital and College of Medicine, Chang Gung University, No. 5, Fuxing St., and No. 259, Wenhua 1st Rd., Guishan District, Taoyuan City 333, Taiwan, China; Center for Vascularized Composite Allotransplantation, Chang Gung Memorial Hospital, No. 5, Fuxing St., Guishan District, Taoyuan City 333, Taiwan, China; Department of Plastic Surgery, Addenbrooke’s Hospital, Hills Road, Cambridge CB2 0SP, UK

**Keywords:** Extracellular vesicles, Engineered EVs, Pluripotent stem cells, Cell-free medicine, Regenerative medicine

## Abstract

The engineered extracellular vesicles (EVs) derived from pluripotent stem cells (PSCs) are a new concept in regenerative medicine. These vesicles are secreted from the embryonic stem cells as well as the induced PSCs (iPSCs) and are involved in the transfer of bioactive molecules required for cell signaling. This review describes the possibilities for their use in the modification of therapeutic approaches in regenerative medicine and targeted therapies. PSCs can differentiate into various cell types that can be useful for tissue engineering or to generate models of diseases in a dish. Compared to cell therapies, engineered EVs are characterized by lower immunogenicity, higher targetability, and improved stability. Some of the applications are angiogenic, tissue restorative, immunomodulatory, and gene therapies for the treatment of certain diseases. iPSC-derived engineered EVs find application in regenerative medicine, drug delivery systems, diagnostics of diseases, and hydrogel systems. In regenerative medicine, they can promote the restoration of cardiac, bone, cartilage, and corneal tissues. Engineered EVs are also employed in drug targeting to particular sites as well as in the diagnosis of diseases based on biomarkers and improving image contrast. Hydrogels that contain EVs provide a depot-based delivery system to slowly release drugs in a controlled manner that enhances tissue repair. Thus, the results described above demonstrate the potential of engineered PSC-EVs for various biomedical applications. Future work will be directed toward expanding the knowledge of engineered PSC-EVs and their possibilities to create new therapeutic approaches based on the functions of these vesicles.

## Graphical Abstract

**Graphical Abstract:**
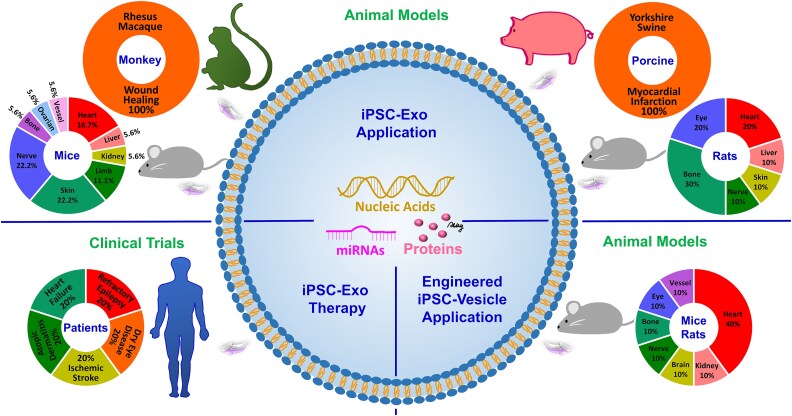

Highlights

- Induced pluripotent stem cell (iPSC)–derived exosomes have shown significant therapeutic potential across various disease models, such as improving cardiac recovery and reducing cell death in heart disease, showcasing their regenerative capabilities.
- Ongoing trials investigate iPSC-derived exosomes for treating epilepsy, stroke, and dry eye, highlighting their clinical relevance.
- Engineered vesicles derived from iPSCs, when integrated with advanced delivery systems like hydrogels and magnetic labeling, have shown enhanced therapeutic efficacy by ensuring sustained release, targeted delivery, and significant improvement in treatment outcomes in various models, including myocardial infarction and corneal regeneration.

## Background

Pluripotent stem cells (PSCs) have revolutionized the field of regenerative medicine and biomedical engineering by presenting unparalleled capability to address imminent medical challenges [1]. The hallmark feature of PSCs is their dual capability: being able to self-renew indefinitely or differentiate into one of the ~200 different cell types in the human body. Thanks to this unique versatility, new opportunities for therapeutic applications and research that were impractical just a couple of years ago are now possible. There are two primary categories of PSCs: embryonic stem cells (ESCs) and induced PSCs (iPSCs). ESCs are derived from the inner cell mass of a blastocyst during the embryonic development processes. These cells inherently have pluripotency and have been a resource for analysis of early human development and tissue differentiation. While widespread application of this approach has been hampered by ethical questions about the embryos, researchers are now searching for other fixes. The final result of this search was the revolutionary finding of iPSCs by the group of Shinya Yamanaka in 2006 [2] with iPSCs made by reprogramming skin fibroblasts from an adult into a pluripotent one via the insertion of a set of key transcription factors Oct4, Sox2, Klf4, and c-Myc [3]. Besides circumventing ethical controversies, this technology also offered a pathway to create patient-specific pluripotent cells, iPSCs, that held the donor’s genetic constitution and are a valuable asset for personalized medicine and autologous therapy. In the development of iPSCs, scientists can model individual patient diseases, test drug responses in the petri dish, and develop customized treatment approaches with a significantly lower risk of immune rejection [4]. PSCs have an impact much greater than the use for cell therapies. PSCs may be used for disease modeling *in vitro* to provide windows to the molecular bases of complex diseases, such as neurodegenerative disorders, cardiovascular anomalies, or genetically determined syndromes. Due to these models, potential therapeutic targets and precision drugs have already been accelerated in the discovery and development phases. Similarly, PSCs have been used to drive forward tissue engineering and organoid technology [4]. Researchers differentiate PSCs into 3D tissue constructs that mimic the structure and function of human organs and are getting closer and closer to the reality of lab-grown transplantable tissue. Additionally, PSCs are instrumental in drug discovery and toxicity testing. The ability to differentiate into cell types such as hepatocytes, cardiomyocytes, and neurons gives pharmaceutical companies a more accurate platform to evaluate the safety and effectiveness of new compounds without using animal models and allowing for more accurate outcomes in human trials [1,5]. However, the PSC-derived cells may be inherently tumorigenic, and immunogenic and are heterogeneous, creating significant challenges for clinical applications. (1) The use of PSC-derived cells may have tumorigenicity and safety concerns. The use of PSCs in cell therapy may have a risk of tumor formation notably teratoma due to uneliminated undifferentiated cells [1]. The risk to grafts is a major barrier to the widespread clinical deployment of PSCs. (2) PSCs may cause immune rejection if allogeneic. PSC-derived cells can undergo transplantation but may induce a host immune response, particularly in the case where cells are not autologous [1]. (3) PSC-derived cells may be heterogeneous and present scalability problems. PSC-derived cells are not easy to maintain because heterogeneity may develop during the expansion and differentiation processes. Standardization and scale-up required for clinical applications are complicated by this variability.

With these challenges in mind, attention has turned to cell-free therapeutic approaches, as extracellular vesicles (EVs) are gaining attention as an appealing alternative [6]. EVs are nano-scale, membranous vesicles released from nearly all cell types. Mediating intercellular communication, they carry bioactive molecules like RNA, proteins, and lipids. These vesicles hold particular promise for regenerative medicine because they mimic the paracrine effects of their parent cells without the risks associated with live cell therapies.

(1) Cell-free nature eliminates tumorigenicity. EVs derived from PSCs provide PSC therapeutic benefits without tumor formation risks. The use of encapsulated bioactive molecules within EVs can lead to regenerative outcomes in a safer, cell-free form.

(2) EVs exhibit lower immunogenicity than cell therapies. In other words, EVs are not as likely as their parent cells to trigger an immune response. Studies have established that PSC-derived EVs can also tone down inflammation, alter the immune system, and induce tissue repair [7].

(3) EVs provide targeted delivery and functional potency. EVs from PSCs are packed with bioactive contents, including growth factors, RNAs, and proteins, to orchestrate their actions in complex regenerative processes. They can engineer or selectively modify their cargo in order to increase the potential for targeting and therapeutic specificity.

(4) EVs are scalable and consistent. Unlike the challenges of maintaining the pluripotent nature of live PSCs during expansion, EV production can be standardized and scaled. The ability of PSCs to be cultured in bioreactors and accordingly, the production of large, uniform quantities of EVs of consistent quality, allows them to meet the demands of the clinic.

(5) Cell-free therapies address ethical and practical issues. PSC-derived EVs avoid ethical issues associated with ESCs as well as technical barriers in the generation of iPS cells. Moreover, EVs are cell-free and, therefore, easier to store, transport, and administer than live cells.

(6) PSC-EVs demonstrate broad therapeutic potential. PSC-derived EVs influence multiple pathways due to their pluripotent origin. For instance, they have the ability to stimulate angiogenesis, and immunoregulation as well as to direct tissue repair within particular tissues, in many preclinical models.

Despite the inherent advantages of PSC-derived EVs, their natural production is typically low, necessitating strategies to enhance yield and functionality. To date, improved cargo loading efficiency, targeting specificity, and therapeutic efficacy, advanced bioengineering approaches such as modulating parental PSCs or modified EVs have been reported [8,9]. Unlocking EVs’ full potential as a regenerative medicine platform will depend on methods including integrating RNA-based payloads, surface functionalization, and scale-up techniques to generate EVs [10]. PSC-derived EVs represent a transformative step in regenerative medicine by bridging the gap between the complex biology of live cell therapies and the safety and scalability of acellular approaches. Utilizing the paracrine effects of PSCs without risk to the cells can open a path for new treatments, as it promises new treatments that circumvent the limitations of both traditional cell-based and nonstem cell-EV applications. In this review, we focus on the PSC-derived EV engineering and therapeutics applications and how these are moving the field forward in developing cell-free regenerative strategies.

## Review

### Pluripotent stem cells

#### Embryonic stem cells

ESCs are generated from the inner cell mass with an early embryonic developmental stage, known as the blastocyst. These cells are naturally pluripotent, which means they possess the inherent capacity to differentiate into any cell type within the three germ layers: ectoderm such as skin and neurons, mesoderm such as muscle and bone, and endoderm such as liver and pancreas. With this exceptional self-renewal and pluripotency, ESCs have become a nucleus of regenerative medicine through the provision of a renewable source of cells to differentiate to the specific types of cells that are required to repair tissues that are damaged or to develop models of organs or to investigate the mechanisms of differentiation [11,12]. The hallmark of ESCs is that they are capable of indefinitely self-renewal while remaining undifferentiated. Together, this attribute with these cells enables researchers to expand these cells *in vitro* without losing their pluripotent potential. Thus, ESCs are useful in that they are easily scalable for cell mass production, and their applications for the mass production of cells interest in drug screening, disease modeling, and transplantation therapies. Because they have a capacity for self-renewal, this produces a steady supply of progenitor cells which can be differentiated into large quantities of cells for many different therapeutic purposes. Also, ESCs offer a window into early human development. Researchers’ ability to study ESCs can aid in understanding the regulatory pathways central to cellular differentiation, organogenesis, and how embryogenesis is governed at the molecular level [13,14]. These studies have greatly enhanced our comprehension of human biology and are helping to form new methodological approaches to congenital disorders. Additionally, ESCs are highly efficient in differentiation protocols. Their robust and reliable pluripotency is in contrast to that of other stem cell types, which can be routinely induced to generate particular cell lineages under defined conditions. Due to this efficiency, they have the potential to be widely used for regenerative medicine research and preclinical studies.

Although great potential to make use of ESCs exists, the utilization of ESCs is not without its difficulties [1]. Obtaining ESCs is associated with a major ethical problem, namely, the destruction of human embryos. As a result, many countries have seen extremely intensive ethical debates and mandated restrictions on ESC-based research and therapies. One more profound limitation of ESC-derived cells is the risk of immune rejection upon use in transplantations. The problem with ESC-derived cells is that they are typically derived from donor embryos, meaning that he or she will have a different set of genes than you, the recipient. The mismatch may provoke immune responses and result in graft rejection and the need for immunosuppressive therapy with its own risk and complications. In addition, ESCs are teratogenic and safety is a risk. Residual undifferentiated cells, if not fully differentiated before transplantation, may form teratomas, tumors containing cells from different germ layers. Thus, ensuring the complete differentiation of ESCs is therefore critical to their safe use in clinical applications.

#### Induced pluripotent stem cells

iPSCs are a revolutionary advancement in the field of stem cell biology, pioneered by Shinya Yamanaka. iPSCs are not directly isolated from ES cells; rather, they are generated from the reprogramming of adult somatic cells such as skin, blood cells, etc., to a pluripotent state induced by the expression of specific transcription factors like Oct4, Sox2, Klf4, and c-Myc. Not only does this breakthrough avoid the ethical controversies surrounding ESCs, but it also allows for the creation of patient-specific pluripotent cells that are genetically identical to the donor and hold many of the characteristics of ESCs, including the ability to differentiate into any of the body’s cell types, with some distinct advantages [2,15]. It has one great benefit: the possibility of personalized medicine. iPSCs are derived from a person’s own somatic cells—the cells within their body—and avoid the immune rejection risk that often follows an allogeneic transplant. Moreover, iPSCs can generate customized therapies that precisely match the genetic and pathological information of each of its patients. iPSCs have massive potential applications. They are highly valuable for regenerative medicine because they can differentiate into specific cell types to regenerate damaged or diseased tissues and are used to treat conditions ranging from neurodegenerative diseases to cardiovascular dysfunction [16,17]. They have been largely instrumental in disease modeling, in which human diseases are recreated *in vitro* for pathophysiology studies and treatment testing [18,19]. In addition, iPSCs are applied in drug discovery and toxicity testing, since they can be differentiated into various cell types and, hence, can give the near-physiological environment for assessment of the safety and efficacy of new compounds.

Finally, though they present with tremendous potential to transform clinical therapy, iPSCs come with challenges, too [3]. Concerns about their safety for clinical applications arise from the possibility that the reprogramming process may introduce genetic and epigenetic abnormalities. Moreover, the efficiency of reprogramming and differentiation kits can be widely variable to the extent that further protocol optimization may need to be performed to achieve reproducibility and scalability. However, the emergence of iPSCs has expanded the regenerative medicine and biomedical research toolbox dramatically and offers new routes for attacking previously intractable medical problems.

### Pluripotent stem cell differentiation

How PSCs develop into a particular type of cell is a very elaborate process that is akin to embryonic development in its complexity. The only cell types that have the ability to give rise to various predetermined cell lineages are ESCs and iPSCs. The difference-making of this process is regulated by molecular events, gene expression, and signaling pathways. The process of differentiation starts with either turning on or turning off the very transcription factors that regulate the cell’s decision-making mechanisms. They work in a similar manner to turn the PSCs toward the lineage specification. Differentiating cells alter their morphology and proteome and the gene expression program of the cells becomes more and more similar to that of the target cell type. This process is ongoing and entails the sequential activation of certain genes that are associated with the choice of lineages of cells with the genes that are associated with stem cells being progressively turned off. The research paper by Laurie A. Boyer and associates provides the details of the key transcriptional network of human embryonic stem cells while focusing on the key elements of OCT4, SOX2, and NANOG [20]. These transcription factors are very important when it comes to supporting the self-renewing capacity of ESCs and the pathways for their differentiation.

Intracellular signals and intercellular communication that are stimulated during this process will continue to be important throughout the process. The signals from the microenvironment that determine the differentiation pathway are the signaling molecules, growth factors, and cytokines [21]. These signals are received by cells through very complex signaling pathways that, in a way, prompt a chain of events that results in either the activation or the inhibition of certain genes. These signaling rates are thus important in terms of their spatial and temporal appropriateness so that the differentiation process can be effective and precise. Here are some examples describing the factors involved in the differentiation of PSCs into various somatic cells. Table 1 below summarizes key differentiation approaches made with PSCs into specific somatic cell types by outlining the factors and methodologies used in these processes. A comparative outlook on *in vivo* and *in vitro* differentiation methods is given in Table 2, comprising their environment, precision, applications, and limitations.

**Table 1:** Key differentiation approaches of pluripotent stem cells into specific somatic lineages

**Differentiation type**	**Example**	**Factors involved**
**Cardiomyocyte differentiation**	Directing human PSCs (hPSCs) into functional cardiomyocytes	Wnt signaling modulators (GSK3 inhibitor, β-catenin shRNA)
**Intestinal tissue differentiation**	Mimicking embryonic intestinal development to form intestinal tissues	Growth factors: activin, FGF, Wnt (WNT3A, FGF4)
**Pancreatic β cell differentiation**	Inducing lineage specification via cytoskeletal modifications	Actin cytoskeleton modulators (latrunculin A)
**Neuronal differentiation**	Generating specific neuronal subtypes	Transcription factor NGN2
**Osteoblast and endothelial differentiation**	Differentiation into osteogenic and endothelial lineages	BMP-2 (expressing markers like osteocalcin, osteopontin, and VEGF receptors)
**Pancreatic lineage differentiation**	Using transcription factors delivered via CPPs	Pdx1, NeuroD, MafA
**Astrocyte differentiation**	Producing astrocytes through neural differentiation or transdifferentiation	Neural transcription factors and small molecules targeting astrocyte-specific pathways

**Table 2:** Comparison of *in vivo* and *in vitro* differentiation methods in pluripotent stem cells

**Feature**	***In vivo* differentiation**	***In vitro* differentiation**
**Environment**	Occurs within the natural developmental setting of an embryo	Mimics embryonic development using controlled culture systems
**Signaling precision**	Naturally coordinated by spatial and temporal signals	Requires precise supplementation of growth factors, cytokines, and extracellular matrix components
**Applications**	Insights into natural development and lineage commitment	Used for regenerative medicine, disease modeling, drug testing, and production of transplantable cells
**Limitations**	Limited accessibility and ethical concerns regarding embryo use	Requires optimization of differentiation protocols to ensure reproducibility and functional maturity of differentiated cells

(1) Cardiomyocyte differentiation: Wnt signaling modulators were used to effectively direct human PSCs (hPSCs) to differentiate into cardiomyocytes [22]. The process involved the use of a GSK3 inhibitor with β-catenin shRNA or Wnt inhibitor for a short time, which resulted in the differentiation of the cells to functional cardiomyocytes.

(2) Intestinal tissue differentiation: Those human PSCs were differentiated into intestinal tissues by the method of mimicking embryonic intestinal development by adjusting growth factors. These were activin-induced definitive endoderm formation, fibroblast growth factor (FGF)/Wnt posterior endoderm patterning, hindgut specification, and morphogenesis; and the prepromulge intestinal culture medium containing WNT3A and FGF4, which were the major regulators [23].

(3) Pancreatic β-cell differentiation: The findings stated that the state of the actin cytoskeleton is responsible for the expression of the pancreatic transcription factors that, in turn, lead to lineage specification. When latrunculin A, which affects actin polymerization, was incorporated during the endocrine induction process, the hPSCs were able to generate pancreatic β cells with improved function [24].

(4) Neuronal differentiation: The process of using transcription factor neurogenin-2 (NGN2) to generate quick and specific differentiation of PSCs into neuronal subtypes is also in use [25]. This proves the significance of NGN2 in neuron differentiation as evidenced by its application in neuronal development and disease modeling.

(5) Osteoblastic and endothelial cell differentiation: In the present study, the NEDAPS (nerve-derived adult PSCs) were induced to differentiate into two lines of stem cells osteogenic and endothelial stem cells. The differentiation process was initiated by the use of bone morphogenetic protein 2 (BMP-2) where cells expressed specific markers such as osteocalcin, osteopontin, and vascular endothelial growth factor (VEGF) receptors [26].

(6) Pancreatic lineage differentiation: Previously, it has been demonstrated that the delivery of proteins by cell-penetrating peptides (CPPs) can induce differentiation of iPSCs into pancreatic lineage. This method involved the use of transcription factors such as Pdx1, NeuroD, and MafA, which were crucial for the application of a nongenomic procedure to differentiation [27].

(7) Astrocyte differentiation: The generation of astrocytes from PSCs involved guided neural differentiation to multipotent neural progenitors as well as transdifferentiation of somatic cells into a fully functional astrocyte. Certain transcription factors and small molecules were used to target the differentiation into astrocytes [28].

However, another factor can be cited and that is epigenetic modifications that greatly influence the differentiation of PSCs. These include DNA methylation modification, histone modification, and chromatin remodeling, which occurs in this process in a way that determines the availability of genes and helps in the setting and stabilization of a differentiated cell state [29–34]. Epigenetic modification offers a way through which the identity and the role of the cell can be maintained when the cell divides. The distinctions of differentiation may result in the specialization of cells into neurons, cardiomyocytes, hepatocytes, and other cell types. The specificity of the lineage during differentiation is clear, which shows how precise and versatile the PSCs are. In addition, the potential of directing differentiation makes it possible to use them for multiple purposes in clinical use like generating transplantation material for therapy, disease modeling, and drug development.

### Advantages of cell-free therapy with extracellular vesicles

**Figure 1:**
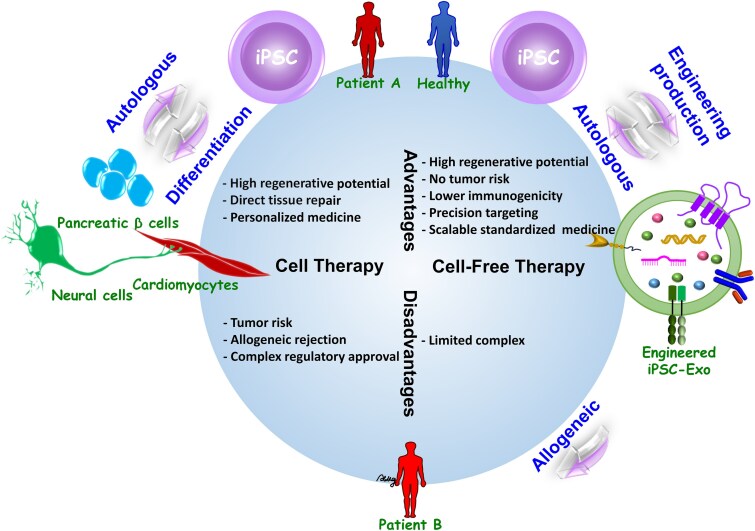
Comparison of cell therapy and cell-free therapy derived from iPSCs. This figure illustrates the comparative advantages and disadvantages of cell therapy and cell-free therapy (engineered iPSC-derived exosomes) derived from iPSCs. The cell therapy is shown on the left, with high tissue regeneration potential, direct tissue repair and the possibility of personalized medicine. However, it is complicated by tumor risk, the potential for allogeneic rejection, and complex regulatory hurdles. On the right, cell-free therapy demonstrates advantages including no tumor risk, lower immunogenicity, precision targeting, and scalability for standardized medicine. However, it has relatively low complexity compared to cell therapy. The diagram highlights the distinction between autologous and allogeneic approaches, as well as the engineering processes involved in creating exosome therapy, offering insights into their clinical applications

Cell-free therapies using EVs from PSC are a groundbreaking alternative in the field of regenerative medicine. Despite the fact that autologous iPSC therapy does not raise the issue of immune rejection when directly applied to patients, the problem of teratoma formation remains. Despite the fact that the use of personalized treatment can overcome the issue of immune rejection, which is always encountered when differentiating PSCs and then allogeneic transplantation, this is rather a very costly approach and the iPSC line is not easily usable by others. If the exosomes or vesicles derived from the stem cell are manufactured, the cell-free treatment is potentially more broadly applicable to patients followed by the different criteria for iPSC therapy. Figure 1 compares cell therapy and cell-free therapy from iPSC, and their respective advantages, disadvantages, and potential clinical applications are shown, as well as the differences between the autologous and the allogeneic approach. The benefits include the following points.

#### Reduced risk of tumorigenicity

Given their major potential for use in cellular therapies, the major risk in the use of PSCs, whether embryonic or induced, is the risk of tumorigenesis, such as the formation of teratoma owing to the remaining undifferentiated cells. Unlike EVs, which are acellular and, as such, not capable of proliferating, these risks do not exist at all. PSC-derived EVs encapsulate bioactive molecules that mimic the therapeutic effects of their parent cells without the possibility of uncontrolled cell growth. Both of these factors make EVs safer for transplantation and regenerative applications.

#### Lower immunogenicity

Live cell therapies, particularly those involving allogeneic iPSCs, have the risk to provoke immune responses, requiring lifelong administration of immunosuppressive regimens that can have significant side effects such as opportunistic infection and tumor formation risk. On the other hand, EVs are by nature less immunogenic and can escape immune detection. Furthermore, studies have shown that PSC-derived EVs can modulate the immune response, reduce inflammation, and promote tissue repair, making them eligible for allogeneic transplantation and wide clinical use without immune rejection complications [7].

#### Targeted delivery and therapeutic specificity

Delivering therapeutic agents with EVs offers a special platform that takes advantage of their natural propensity to mediate intercellular communication. Specifically, the ability of these particles to carry bioactive molecules—nucleic acids, proteins, lipids, and growth factors, and deliver them to specific cells or tissues—is unique. The biological properties of EVs can be used to deliver drugs, however, their targeting specificity and therapeutic efficacy can be further enhanced by modifying or engineering them.

##### Natural targeting capabilities

Surface markers and membrane proteins inherited by EVs from the parental cells enable them to target specific recipient cells. Examples of this include tumor-derived EVs that utilize the expression of integrins or adhesion molecules on their surface, such that they will preferentially target tumor cells [35]. It is known that immune cell-derived EVs can naturally be home to sites of inflammation or injury [36,37]. The targeting ability of EVs therein is natural, which makes EVs very efficient for therapeutic applications minimizing the requirement for external modifications and biological compatibility maintained.

##### Engineered targeting for enhanced precision

Natural EVs already show some targeting; however, this is significantly increased through engineering techniques such as surface functionalization, receptor–ligand engineering, and pH or enzyme-sensitive release systems. Specific cell surface markers can be bound to EV membranes by modifying them with ligands, antibodies, or aptamers. For instance, an EV can be functionalized with antibodies to HER2 so that the EV is selectively targeted with therapeutic agents to HER2-positive breast cancer cells [38–40]. Furthermore, EVs can be engineered to express ligands that will bind to the receptors on target cells, thereby increasing their ability to localize and deliver therapeutic cargo to diseased tissues [41,42]. Moreover, EVs also can be designed to release their contents in response to the microenvironment of the target tissue, such as the acidic pH of tumors or specific enzymatic activity in inflamed tissues [43,44]. These engineering strategies overcome this delivery challenge by delivering therapeutic molecules precisely where they are needed, minimizing systemic exposure and off-target effects.

##### Therapeutic cargo customization

EVs were found to be able to transport a wide variety of therapeutic agents, including nucleic acids, proteins, peptides, and small molecules that can be selectively loaded to meet the needs of a particular treatment. With EVs, small interfering RNA (siRNA), microRNA (miRNA), or mRNA can be delivered to target cells for regulating gene expression—a therapeutic option for genetic disorders and cancer. In addition, EVs can be loaded with therapeutic peptides or proteins for function modulation or damaged tissue repair. Additionally, EVs can be encapsulated to achieve small-molecule drugs to enhance their stability, bioavailability, and delivery efficiency. Customizable EV cargo gives unrivaled flexibility for developing targeted therapies for most diseases.

##### Enhanced therapeutic efficacy

Not only does this delivery reach the target site, but it also increases the efficacy of the therapeutic agents by maximizing the treatment at the site of disease or injury. Their localized delivery can increase the potency of the therapeutic effect by minimizing the dosage needed, thus reducing potential side effects. This simultaneously improves the safety profile of the therapy, removing the possibility of unintended interactions with nontarget tissue.

###### Overcoming biological barriers

In contrast to traditional drug delivery systems, EVs naturally address limitations such as the blood–brain barrier (BBB), immune escape, and tissue penetration. Therefore, these EVs can cross the BBB and are therefore ideal for treating neurological diseases such as Alzheimer’s disease, Parkinson’s disease, and glioblastoma. Furthermore, EVs can evade immune detection and clearance, allowing efficient delivery to target cells without eliciting an immune response. In addition, EVs have nanoscale dimensions that can be used to penetrate deep into tissues and enter areas inaccessible to larger therapeutic delivery systems.

###### Clinical applications of targeted EV therapies

In preclinical and clinical applications including cancer therapy, regenerative medicine, and autoimmune diseases, EVs have been demonstrated to possess targeted delivery and therapeutic specificity [7]. Alternatively, EVs engineered to transport chemotherapeutic drugs or genetic material can be directed to specifically target tumor cells, thereby decreasing off-target toxicity and increasing the efficacy of treatment [10,45,46]. Furthermore, EVs loaded with growth factors or stem cell–derived signals can enhance cardiac, neural, and musculoskeletal tissue repair and regeneration in injuries [47]. In addition, EVs can deliver immune-modulating molecules specifically to immune cells and do so to restore immune balance in rheumatoid arthritis and multiple sclerosis [48,49].

##### Future directions in targeted EV therapies

With further research, the targeted delivery enabled by EVs such as artificial EVs, combination therapies, and real-time tracking will be improved. Bioengineered or synthetic EV-like nanoparticles can be designed to mimic natural EVs with enhanced targeting and cargo-loading capabilities. Furthermore, EVs in combination with other therapies, such as immunotherapy or radiation, facilitate their synergistic effects. In addition, imaging agents can also be loaded into EVs to enable real-time tracking of distribution *in vivo* and such therapeutic effects.

#### Scalability and consistency

Maintaining the pluripotent state of live PSCs and providing consistent differentiation outcomes are often hindered by challenges to their production. On the other hand, EVs can be manufactured in large quantities based on similar protocols. The ability to culture PSC in bioreactors to produce EVs of consistent quality and functionality to meet clinical-grade requirements is achievable. EVs have the scalability that makes them a viable solution for widespread therapeutic use.

#### Enhanced stability and storage

EVs are more stable and easier to store and transport than their live cell counterparts, which necessitate stringent conditions for viability. Given they can be lyophilized or cryopreserved without significant loss of functionality, they are perfectly suited for distribution to a wide range of healthcare settings. This logistical flexibility greatly mitigates the problems related to cell-based therapies and promotes the accessibility of EV-based treatments.

#### Addressing ethical and practical concerns

Derivation of ESCs raises ethical issues in the destruction of the embryos while the production of iPSCs is laborious, technically complex, and expensive. Concerns surrounding this field may be circumvented by cell-free therapies using EVs. EV production, which involves no destruction of embryos, or the reprogramming of somatic cells, is more acceptable ethically. Furthermore, EV therapies also simplify potential regulatory pathways as they work with acellular products as opposed to live organisms.

#### Broad therapeutic potential

The pluripotent origin of PSC-derived EVs makes them uniquely versatile. Bioactive molecules that can influence multiple regenerative pathways, such as angiogenesis, immune modulation, and tissue-specific repair, also carry. PSC-EVs can promote the formation of new blood vessels, critical for wound healing and tissue repair and reduce inflammation and enhancing immune regulation in autoimmune and inflammatory conditions. Additionally, PSC-EVs also can guide the regeneration of specific tissues such as neural, cardiac, or hepatic cells. Such broad applications indicate the use of EVs to overcome complex and systemic conditions for which traditional therapies do not provide effective solutions.

### Extracellular vesicle origin and biogenesis

The subject of EVs coming from PSC biogenesis has, in the recent past, received a lot of focus because of its relevance in the area of stem cell therapies. Both ESCs and iPSCs are PSCs that release EVs as a means of cell–cell communication. EVs consist of compartments derived from the cell, and their assembly is associated with the key cellular processes [50–52]. The smallest of the EVs are the exosomes which are formed within a multivesicular body (MVB) during the process of endocytosis of a cell. Microparticles or shedding vesicles are produced when vesicles bud out and there is the fission of the plasma membrane. Instead, apoptotic bodies are formed because of the segmentation of the cells that are undergoing programmed death or apoptosis [53]. Genomic profiles of each subtype that depict the source of the cell and the processes that led to the development of the subtype are different in each subtype.

**Figure 2:**
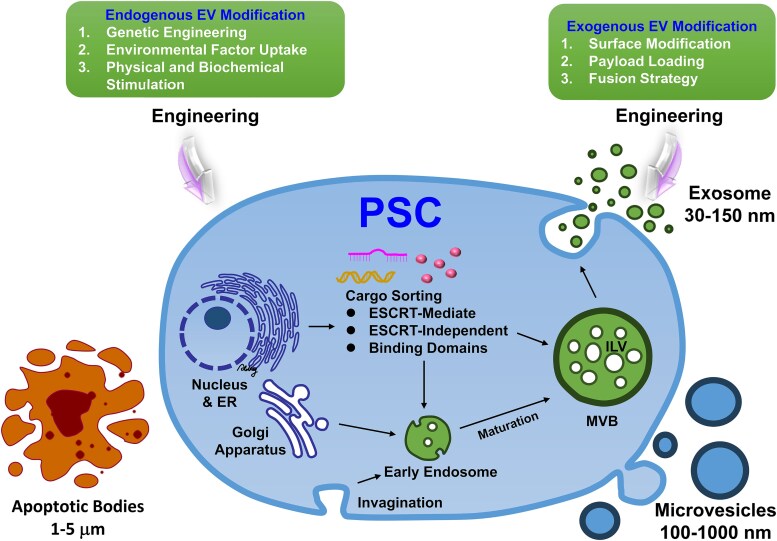
Engineered PSC-derived extracellular vesicle biogenesis and engineering strategies. The biogenesis of EVs produced by PSCs is depicted in this figure, as are engineering strategies to improve EV functionality and therapeutics. It starts inside PSCs with the sorting of cargo through ESCRT-dependent and independent mechanisms and with binding domains. Endosomes mature early and become multivesicular bodies (MVBs) that contain ILVs as exosomes 30–150 nm in size, or budding microvesicles of 100–1000 nm at plasma membrane. Apoptotic bodies (1–5 μm) are formed during programmed cell death. The **‘**engineering strategies’ are divided into two categories based on the target of modification: (1) ‘endogenous modifications’, where parent cells such as PSCs are treated to result in delivering different types of EV, cargo, and biogenesis. Optimization of the EVs before they are released, includes genetic engineering, uptake of environmental factors, and physical or biochemical stimulation of the cells to release EVs. (2) ‘Exogenous modifications’, which are directly applied to the EVs after their release, include surface modification, payload loading, and fusion strategies. Both types of engineering strategies enhance the targeting specificity, stability, and therapeutic applications of EVs

(1) Exosomes, which are the smallest of the EVs, originate from the endocytic pathways and especially from MVBs. These are features that are demonstrated by proteins like Alix and TSG101 that are associated with the endosomal sorting complex required for transport (ESCRT) complex system. Also, exosomes contain several tetraspanins (CD9, CD63, and CD81), heat shock proteins, and lipids and this suggests that exosomes are derived from endosomal biogenesis and are involved in the immune system and cell signaling.

(2) Microvesicles, which are larger than exosomes, are directly outward budding and fission of plasma membrane. These vesicles also contain cell-specific markers like integrins and selectins which are markers of cell surface. Microvesicles may contain cytoplasmic components such as enzymes and signaling molecules because of the manner in which they are generated as opposed to exosomes. This could be because they have more surface receptors and adhesion molecules; hence, they are involved in intercellular adhesion, coagulation, and inflammation.

(3) Apoptotic bodies, which are the largest among EVs, are released to the extracellular space during apoptosis. These are phagosomes that surround cellular debris and organelles and have phosphatidylserine on the surface of their membrane. The apoptotic bodies are recognized by the nuclear and cytoplasmic contents that they enclose; this gives a clear message about the function of the apoptotic bodies in the clearance of the apoptotic cells and cellular debris. Apart from this, apoptosis is also associated with caspase activation and cleavage of cytoskeletal/nuclear proteins, which result in the generation of apoptotic bodies that are distinguished by certain molecular signatures. Thus, disputing over the molecular characteristics of exosomes, microvesicles, and apoptotic bodies could have been the indication of their different origins besides the fact that they serve different functions. Despite the fact that exosomes are thought to be derived from endosomes, microvesicles have their characteristic surface markers, and apoptotic bodies contain a mixture of proteins involved in the cell death pathway. Thus, by understanding these molecular differences, it is possible to enhance the ability to harness EVs in diagnostics, therapeutics, and signaling. The biogenesis of PSC-EVs is illustrated in Figure 2, and strategies for engineering the EV cargo using endogenous and exogenous modifications for enhanced therapeutic potential are discussed.

The process of formation of vesicles for secretion is a complex process that occurs inside the parent cells [50,54]. One such process is the intracellular formation of EVs within the endosomal compartment. Given that PSCs have the potential of differentiating into various cell types, they ensure that bioactive cargo is sorted into different subsets of EVs. At this step, the endosomal membrane invaginates to form the early endosomes. Through this process, some of the proteins, lipids, and nucleic acids are sorted out and encapsulated in the form of intraluminal vesicles (ILVs). This process known as inward budding is done in the context of the ESCRT machinery, which is a protein complex that is in charge of cargo recognition as well as vesicle formation. Subsequently, the early endosomes mature into the late endosomes where lipid vesicles otherwise known as ILVs are formed with a combination of proteins, nucleic acids, and other bioactive molecules as the load. The maturation process is succeeded by a more elaborate sorting process so as to correctly sort in or out the specific cargo of these vesicles. The other important factor is the endosomal system, which not only regulates the process of EV secretion but also interacts with other cellular pathways, which gives a possibility for the adaptive response to the microenvironment and influences the composition of the EVs. The final process in the formation of EVs is when ILVs are released in the extracellular space as either exosomes or microvesicles that are derived from the plasma membrane. This process is regulated by many molecular activities including the fusion of MVBs with the plasma membrane. At other times, microvesicles are secreted directly in the extracellular space. Thus, the EVs produced are a reflection of cargo defined by the parental PSC identity and differentiation status, as well as by their sensitivity to the environment.

### Extracellular vesicle characteristics

EVs are a rather diverse type of cell-derived structures that have been recently increasingly recognized as extracellular vectors in various cell signaling and homeostatic processes. The vesicles described here such as exosomes, microvesicles, and apoptotic bodies have a number of properties that are more diverse than the preceding vesicles, which shows their importance in cellular communication and their potential use in diagnostics or therapy.

#### Size

EVs are a heterogeneous group of membrane-bound particles secreted by cells, varying widely in size [55]. This variability in size is not simply a size variable but is integral to their function and to the diverse ways they might be applied biologically. Their cargo capacity, cellular interaction mechanisms, and functional roles depend on the EV range in size. (1) Exosome size ranges from 30 to 150 nm, which is the smallest as compared to other EVs. They come out of the endosomal pathway, where they form ILVs within the MVBs. Exosomes are released into extracellular space, following the fusion of MVBs with the plasma membrane. Their small size makes them effective for communication between cells and their uptake by recipient cells via endocytosis or receptor-mediated pathways is efficient. Their nanoscale size makes them particularly suitable for drug delivery systems and enables them to transport medicines through biological barriers such as the BBB [56,57]. (2) Microvesicles have a larger size with diameters ranging from 100 to 1000 nm. The regulated outward budding and fusion of the plasma membrane form these vesicles, which are controlled by changes in cytoskeleton and lipid microdomain composition. They can transport a larger range of cargo, with a broader cargo capacity by virtue of their larger size, including larger protein complexes, cytoplasmic enzymes, and other signaling molecules. Their size allows them to serve in diagnostic applications where surface marker profiling can provide insights into the state of the parent cell, such as in cancer or inflammation [58]. (3) Apoptotic bodies are the biggest EVs, sometimes even hundreds of micrometers large. During programmed cell death (apoptosis), these vesicles are released as the cell breaks up into smaller components. They comprise cellular debris, organelles, and nuclear fragments all within a membrane. Apoptotic bodies are large enough to haul entire organelles or large protein aggregates, which makes them essential for removing debris and keeping a tissue’s homeostasis. As such they act as a signal for phagocytosis, marking apoptotic cells for engulfment by immune cells such as macrophages [59,60]. Additionally, apoptotic bodies may be exploited in biomarker discovery since they contain the cellular state during apoptosis [61,62].

Therefore, cargo transport: microvesicles and apoptotic bodies transport bulky cargo and can be large (50–1000 nm in diameter), while exosomes are small (50–100 nm in diameter) and specialize in targeting specific, high concentrations of small bioactive molecules. Functional roles: The size affects the ability of EVs to cross biological barriers, interact with target cells, or contribute to other physiological processes such as immunomodulation, angiogenesis, and tissue repair.

#### Composition

EV composition is a heterogeneous population of nanostructures that contain bioactive molecules, most of which are released by the parental cells. The proteomic analyses have indicated that there are numerous proteins that are the main actors in several cellular processes like transport of membranes, signal transduction, and organization of the cytoskeleton [55].

(1) Exosome composition reflects their endosomal origin. Key components include proteins, nucleic acids, and lipids. Typical exosome ‘marker proteins’ are tetraspanin (CD9, CD63, CD81), and ESCRT-associated proteins (Alix and TSG101), involved in exosome biogenesis. Stress response and protein folding, in which ‘heat shock proteins’ like Hsp70 and Hsp90 are involved, are necessary for protein stability. Enzymes, growth factors, and signaling molecules mediating intercellular communication are ‘functional proteins’ contained in exosomes. ‘Nucleic acids (mRNAs and miRNAs)’, transported through exosomes to recipient cells, modulate gene expression in recipient cells in a manner that affects cellular behavior. For example, miRNAs carried within exosomes govern immune responses and progression of a tumor [63,64]. Some exosomes contain fragments of genomic or ‘mitochondrial DNA’ that can function as a biomarker of cellular states or diseases [65,66]. ‘Lipid phosphatidylserine’ in exosomal membranes is engaged in cell recognition and uptake by recipient cells. These lipids like ‘cholesterol and ceramides’ maintain membrane structure and fluidity; they are also involved in the formation of exosomes. Due to their ability to transfer molecular cargo to recipient cells, exosomes contribute to angiogenesis, immune modulation, and tumor metastasis.

(2) The composition of microvesicles reflects their membrane origin since they are generated through outward membrane budding. In addition, proteins, nucleic acids, and lipids are key components of microvesicles (MVs). MVs harbor ‘surface markers’ such as integrins, selectins, and cadherins typical of their importance in cell adhesion and signaling. Many ‘cytoplasmic proteins’ such as enzymes, cytoskeletal proteins (actin and tubulin), and metabolic proteins are often encapsulated in MV due to their cytoplasmic origin. MVs, like exosomes, contain ‘mRNA and miRNA’ and thereby can regulate gene expression in recipient cells. Moreover, compared with exosomes, MVs can package larger fragments of ‘DNA’, reflecting their ability to transport a range of molecular cargo. ‘Membrane lipid’ species including phospholipids, sphingomyelin, and cholesterol are abundant in MV membranes for structural integrity and functional reasons. Closer inspection showed that because ‘lipid rafts containing adhesion molecules’ are present, MVs can join coagulation, immune responses, or intercellular communication. As a result, MVs are implicated in numerous physiological processes, including inflammation, coagulation, and tissue repair [58,67]. Because of their bigger size, they can haul more cargo—such as sophisticated proteins and signaling molecules—and are useful in the mediation of other biological processes.

(3) Apoptotic bodies are formed during programmed cell death (apoptosis) and carry diverse cellular debris. Among constituents, proteins, nucleic acids, and lipids are key components of apoptotic bodies. Apoptotic bodies linked to apoptosis usually include ‘apoptotic protein markers’, including caspases and cleaved cytoskeletal proteins. Proteins also contained within both ‘nuclear and cytoplasmic protein compartments’, such as nuclear fragments, cytoplasmic enzymes, and organelle-specific proteins in apoptotic bodies, reflect the progenitor cell’s composition. Some apoptotic bodies may contain pieces of fragmented ‘genomic DNA and other RNA’ species, reflecting the breakup of the nucleus and cytoplasm during apoptosis. The key marker on the surface of apoptotic bodies, ‘phosphatidylserine lipid’, acts as a signal for phagocytic cells to clear apoptotic debris by engulfing. ‘Lipids’ within the apoptotic body membrane are also the lipids of the plasma membrane and thus impart structural integrity during cellular fragmentation. Consequently, apoptotic bodies are the main repositories for apoptotic debris clearing apoptotic debris and sustaining tissue homeostasis. They can help signal phagocytes or surrounding cells to help modulate immune responses. Recent research is indicating therapeutic applications as stimulators of tissue repair and immune modulation [68].

#### Biological activity

EVs are involved in many biological functions, which demonstrate the functional spectrum of EVs [69–72]. The most important function of EVs is to transport messages from one cell to another cell of the organism. For instance, exosomes are capable of transferring active molecules to target cells, and this influences the occurrence of such events as cell differentiation, modulation of immune responses, and tissue repair. Microvesicles are the larger vesicles and they can be possible carriers of more cargo. They also help in intercellular communication. The activities are based on the subtype of the EVs—exosomes, microvesicles, and apoptotic bodies—and all have distinct roles in the physiological and pathological processes.

(1) Exosome biological activity is tightly linked to their capacity to interact with target cells and modulate signaling pathways. They may predominantly be involved in signaling pathways such as Wnt/β-catenin, PI3K/Akt, MAPK/ERK, NF-κB, Notch, and transforming growth factor beta (TGF-β) pathways [73]. Cancer cell–derived exosomes transport Wnt ligands to recipient cells for activation of the ‘Wnt/β-catenin pathway’ to promote tumor progression, metastasis, and epithelial–mesenchymal transition [74,75]. Growth factors present in exosomes including VEGF and TGF-β enrich exosomes, activate the ‘PI3K/Akt pathway’ in recipient cells, and promote angiogenesis, cell survival, and proliferation [76,77]. Activated by exosomal cargo like oncogenic miRNAs or signaling proteins, the ‘MAPK/ERK pathway’ may induce tumorigenesis and drug resistance [63,78]. Exosomes carrying pro-inflammatory cytokines including IL-6 and TNF-α activate the ‘NF-kB pathway’ in recipient immune cells to amplify immunoinflammatory responses seen in rheumatoid arthritis and inflammatory bowel disease. The Notch signal, given off by exosomes by means of Notch ligands for the ‘Notch pathway’, binds to Notch receptors present on the recipient cells and thus influences cell fate decision and angiogenesis [79–82]. Exosomes may also contain TGF-β for the TGF-β pathway, and other immunosuppressive molecules can modulate immune responses by promoting regulatory T-cell activity [83,84].

(2) Microvesicles at cell injury interact with specific cell surface receptors to fulfill roles in inflammation, coagulation, and tissue repair. Microvesicles can carry integrins such as αvβ3 to act as ‘integrin-mediated signaling’ to influence cell adhesion, migration, and angiogenesis through interacting with extracellular matrix components, or target cells [85–87].

(3) Apoptotic bodies perform these roles primarily if debris is to be cleared or immune modulation is conducted. Apoptotic bodies are generated during apoptosis, and they are involved in the ‘caspase pathway’. The caspase mediates the cleavage of cytoskeletal and nuclear proteins, which signal for clearance of the debris by macrophages and other phagocytes [88,89]. Phosphatidylserine on the surface of apoptotic bodies involved in **‘**phosphatidylserine-dependent signaling**’** acts as an “eat-me” signal, triggering phagocytic engulfment and immune resolution [90,91].

Also, EVs were involved in disease progression including cancer, neurodegenerative diseases, and inflammation.

(1) Cancer-related EVs are involved in tumorigenesis, metastasis, and immune evasion during the course of cancer [92]. Tumor-derived exosomes are very loaded with oncogenic materials such as protein, nucleic acid, and growth factors and thus are involved in the conversion as well as determining the environment that favors neoplastic transformation. Not only that, but EVs directly circulating in the blood may also be employed to serve as cancer biomarkers to gather information concerning the molecular characteristics of the primary tumors.

(2) Alzheimer’s and Parkinson’s diseases, which are neurological disorders, are known to be associated with EV dysregulation [93]. The exosomes released from neural cells contain pathological proteins, some of which include tau and a-synuclein, which are linked with the present neurodegenerative diseases. EVs become a path through which misfolded proteins are transported to the nervous system cells, thus creating a pathogenic condition; the pathogenic condition is then spread to other cells of the central nervous system. Thus, the role of EVs in the development and progression of neurodegenerative diseases’ pathogenesis is disclosed, and possible therapeutic targets are also outlined. Among these functions, in inflammatory conditions like rheumatoid arthritis or inflammatory bowel disease, EVs play a role in immune regulation and the propagation of inflammation as well [93]. Immune cells release EVs, which contain the pro-inflammatory cytokines, thus magnifying the inflammatory response. The EVs also are involved in the immune system cell signaling, especially cell-to-cell communication. Hence, EVs affect the activity of immune cells as well as the course of inflammation and its outcomes. These examples show the different ways of involvement of EVs in the disease process that is going on inside the body. The feature of carrying some kind of cargo and sending messages to particular cells is an essential aspect to recognize EVs in diseases. It gives potential therapeutic targets, diagnostic markers, and detailed information on the molecular basis of various diseases with different features.

In general, it is possible to state that the understanding of the origin, particle size, substance composition, and biological activity of vesicles has significantly expanded, which makes it possible to study their complex functions in the organization of cellular communication and disease development. EVs are scientifically appealing due to their ability to transfer bioactive molecules between different species which is a feature of their heterogeneity. These factors make EVs promising objects of investigation in both basic science and application as well as in clinical use. Here is the summary of this information in Table 3.

**Table 3:** The summary of origins, sizes, compositions, and biological activities of exosome, microvesicle, and apoptotic body

**Feature**	**Exosome**	**Microvesicle**	**Apoptotic body**
**Origin**	Endocytic pathway, within MVBs	Outward budding of plasma membrane	Fragmentation during apoptosis
**Size**	30–150 nm	100–1000 nm	>1000 nm (several micrometers)
**Composition**	Proteins: Tetraspanins (CD9, CD63, CD81), Alix, TSG101, heat shock proteins, signaling proteins, etc. Nucleic acids: mRNA, miRNA, DNA Lipids: Phosphatidylserine, cholesterol, ceramides	Proteins: Cell-specific markers (integrins, selectins), cytoskeletal proteins Nucleic acids: mRNA, miRNA, DNA Lipids: Phospholipids, sphingomyelin, adhesion molecule-enriched	Proteins: Apoptotic markers, nuclear and cytoplasmic proteins Nucleic acids: Fragmented DNA, RNA Lipids: Phosphatidylserine, plasma membrane-derived lipids
**Key signaling pathways**	Wnt/β-catenin, PI3K/Akt, MAPK/ERK, NF-κB, Notch, TGF-β	Integrin-mediated	Caspase, phosphatidylserine-dependent
**Biological activity**	Intercellular communication, immune regulation, cell signaling	Intercellular adhesion, coagulation, inflammation	Removal of apoptotic cells and debris, cellular cleavage processes

### Extracellular vesicle application

EVs derived from various sources offer unique therapeutic potentials, but PSC-derived EVs demonstrate unparalleled advantages across applications due to their pluripotent origins, diverse cargo, and scalability. Among EVs from different sources, PSC-derived EVs exhibit unique advantages over those from nonstem cells and adult stem cells, such as mesenchymal stem cells (MSCs) [47]. In this study, we define MSCs as a population of multipotent stem cells capable of differentiating into mesodermal lineages, such as osteoblasts, chondrocytes, and adipocytes, with self-renewal capabilities. In contrast, the term “mesenchymal stromal cells” refers to a broader and heterogeneous group of cells from the stromal compartments of tissues, which may or may not include cells with stem-like properties.

(1) PSC-EVs have pluripotent origins and broader differentiation potential. Non-stem cell-derived EVs isolated from differentiated cells including endothelial cells, fibroblasts, or immune cells contain low levels of bioactive material that resemble the distinctive functions of their parent cells. These are usually localized signaling and specific functions, for example, angiogenesis in endothelial cells. MSC-EVs show multipotentiality and can differentiate into mesodermal lineages including bone, cartilage, and adipose tissues. Their immune and bone-forming properties are anti-inflammatory and immunomodulatory, which make them particularly suitable for wound healing and osteogenesis. In contrast, PSC-EVs, stemming from pluripotent cells competent to differentiate into all three germ layers, namely, ectoderm, mesoderm, and endoderm cells, transport a more extensive and potent load of biological molecules including pluripotency factors, such as Oct4, Sox2, Nanog, and lineage-specific signals from various tissue types. Consequently, the pluripotent origins of PSC-EVs allow them to offer versatility and open possibilities beyond those offered by MSC-EVs and non-stem cell-EVs for complex tissue repair and systemic regeneration.

(2) PSC-EVs have a rich and diverse cargo profile. The cargo of nonstem cell EVs is specific to their parent cell types (e.g. angiogenic factors from endothelial cells or structural proteins from fibroblasts), limiting their therapeutic applicability. MSC-EVs are chock full of anti-inflammatory cytokines, growth factors such as VEGF, TGF-β, and miRNAs that positively support localized repair as well as limiting inflammation. However, they lack signals for systemic regeneration. In contrast, PSC-EVs secrete a cocktail of bioactive molecules, ranging from developmental regulators to pluripotency-associated RNAs and proteins, capable of enhancing the repair of different tissue types. Additionally, they can promote angiogenesis; guide differentiation into specific lineages such as neural, cardiac, or hepatic; and they can influence cellular reprogramming. The versatility and efficacy of PSC-EV cargo thus permit more general applications, including systemic therapies and complex tissue engineering.

(3) PSC-EVs originate from parent cells with higher proliferative capacity. While nonstem cell EVs are relatively easy to produce, they are often constrained by donor cell viability and inconsistent EV composition. The quality of donor cells is critical to MSC-EV production, which has high variability due to donor age, health status, and culture conditions. Adult tissue–derived MSCs have limited proliferation capacity *in vitro*, and this limited expansion is limiting scalability. In contrast, PSCs can be grown indefinitely in culture under defined culture conditions and EVs produced at a large scale and of high quality. Finally, industrial-scale production is aided by advanced bioreactor technologies. Thus, PSC-EVs offer greater scalability and batch-to-batch consistency than MSC- and nonstem cell–derived EVs.

(4) PSC-EVs have broader therapeutic applications. Nonstem cell EVs are more effective for localized functions, for example, to drive angiogenesis or to deliver specific molecules, and have more limited applicability for systemic or complex tissue regeneration [38]. MSC-EVs can be used especially in wound healing, cartilage repair, and anti-inflammatory applications [94]. However, their regenerative versatility is limited due to the constrained differentiation potential of MSCs. In contrast, PSC-EVs are effective in a wide range of applications, including neural regeneration, cardiac repair, and hepatic regeneration [7]. In preclinical models of ischemic injury, neurodegenerative disease, and organ repair, they have been shown to be more efficacious [95]. Therefore, the broader therapeutic scope and ability to influence systemic regeneration make PSC-EVs distinctively superior to MSC- and nonstem cell–derived EVs.

The usual situation with the wide usage of EVs in regenerative medicine is observed again. EVs derived from PSCs are capable of regeneration and are used in tissue repair and regeneration. These exosomes have growth factors, miRNAs among others that are signals for cells to grow and get differentiated and hence can be used in tissue regeneration and healing of tissues as therapy [96–99]. Some of the growth factors contained in the vesicles are involved in the process of cell growth and tissue repair and, at the same time, start a chain of molecular events that cause repair of affected or pathological tissue. The presence of miRNAs in the PSC-derived EVs further enhances the regenerative capacities of these cells. miRNAs are small endogenous RNA molecules that regulate gene expression and are involved in numerous biological processes. In EVs, miRNAs can be targeted to the cells of interest, which might regulate the gene expression and signaling pathways and therefore improve tissue repair. This particular delivery system enhances the efficacy as well as the result of regenerative medicine, hence allowing a strategic approach to the therapeutic effects. Wang *et al*. showed that iPSC-EVs reduced oxidative stress-mediated cell death *in vitro* for cardiomyocytes and inhibited cell death by caspase 3/7 *in vivo*. Another property of EV is related to the cytoprotective effect, which is also associated with certain miRNAs, namely, miRNA-21 and miRNA-210 [100]. Xuan *et al*. used small molecule ISX-9 to induce cardiac progenitor cells (CPCs) from iPSCs and derived EVs to remodel the heart after infraction as per the densities of miRNAs [101]. For instance, CPCISX-9-derived EXs, which contain upregulated miRNA-520/-373, possessed anti-fibrosis effects by down-regulating TGF-β and hypoxia-induced genes such as GDF-11 and ROCK-2 and enhanced cardiomyocyte proliferation and angiogenesis [102]. This can be used to treat ischemic heart diseases, and this is an enhancement over the limitation of cell-based therapy [102]. Also, a characteristic of PCSC-derived EVs is that they can promote cell growth and development, which is essential for tissue regeneration. Therefore, these vesicles decide the destiny of the recipient cells and present the focused cells required for tissue restoration. This differentiation-inducing capacity is, however, of importance, especially in the field of regenerative medicine in which the goal is to create new tissue to replace the damaged one. For this reason, stem cell–derived EVs can be applied in tissue repair and regeneration therapy due to their therapeutic properties. These EVs can be used in interventions with the purpose of stimulating the process of tissue regeneration in different diseases, injuries, neuropathies, and after surgeries due to their noninvasive nature and the possibility to control cell functions and may be seen as the future of individualized regenerative therapies.

MVs derived from PSCs also demonstrate their potential applications. The main approaches of ESC-derived MVs involve treating diseases like cancer and cardiovascular conditions by employing their strong autophagy and apoptosis-inducing properties [103,104], whereas iPSC-derived MVs are widely employed in regenerative medicine for skin rejuvenation, wound healing, and cardiac repair, by miRNA delivery and extracellular matrix remodeling [100,105,106]. In ESC-derived microvesicle applications, Ji *et al*. demonstrated that ESC-derived microvesicles, particularly from human embryonic stem cell–derived mesenchymal stem cells (hESC-MSCs), have shown antitumor activity [103]. They prevent cell proliferation of leukemia by downregulating the Bcl-2/Bax ratio, upregulating the expression of Beclin-1, and eliciting conversion of LC3-II, thus inducing autophagy and apoptosis. Moreover, Zhang *et al*. demonstrated that microvesicles of human embryonic neural stem cells (hESC-NSCs) could enhance autophagy, decrease apoptosis in HL-1 cardiomyocytes, and restore full functional activity of cardiomyocytes [104]. This is achieved by transporting HSP-70 and activation of the AKT and mTOR pathways, which represent important cellular factors in mitigating myocardial reperfusion injury. In iPSC-derived microvesicle applications, Wang *et al*. indicated that iPSC-derived MVs (iPSCs-MVs) deliver cardioprotective microRNAs such as miR-21 and miR-210 to prevent apoptosis in ischemic cardiomyocytes [100]. These MVs improve myocardial ischemia/reperfusion outcomes and offer a tumor-free alternative to direct iPSC therapies. Yan *et al*. demonstrated that iPSC-derived MVs have demonstrated potential in accelerating second-degree burn wound healing through miR-16-5p, which promotes keratinocyte migration by targeting Desmoglein 3 (Dsg3) and activating the p38/MAPK pathway [105]. That enhances re-epithelialization and tissue repair. In the field of regenerative medicine, Bakhshandeh and colleagues demonstrated that iPSC-derived MVs enhance fibroblast activity and stimulate the production of extracellular matrix proteins (collagen I and III) while promoting dermal fibroblast migration without affecting their proliferation [106]. In addition, they help to form blood vessels, necessary for the repair of tissues.

#### Embryonic stem cell–exosome animal application

The detail of the therapeutic use of the ESC-derived exosomes in different animal models is presented in Table 4. Exosomes are new-generation carriers of regenerative therapies because they can transport bioactive molecules. The following table shows the various disease targets, the origin of exosomes, their size, and the results of the treatment. In cardiac function recovery, ESC-exosomes significantly enhance cardiac function in myocardial infarction and heart failure models. In a mouse model of transverse aortic constriction (TAC)–induced heart failure, for instance, exosomes enhance cardiac function and angiogenesis while preserving microvessel integrity [107]. In liver fibrosis induced by carbon tetrachloride, ESC-exosomes reduce fibrosis and boost liver regeneration [108]. For pulmonary fibrosis in bleomycin-induced models, ESC-exosomes alleviate inflammation and fibrosis, improving pulmonary function [109]. In neural protection, ESC-exosomes derived from neural cells protect against ischemia-induced neuronal damage [110]. oxygen–glucose deprivation (OGD) dramatically depresses survival, leads to apoptotic cell death, and affects synaptic functions, and they alleviate these OGD effects by increasing survival decreasing apoptosis and restoring synaptic functions. In bone regeneration, ESC-exosomes promote differentiation and bone repair, especially in models of osteoporosis and bone defects [111]. Osteogenic markers such as collagen are enhanced, and bone architecture is improved. In anti-inflammatory and pyroptosis inhibition, ESC-exosomes effectively reduce pyroptosis and inflammation in models of doxorubicin-induced cardiotoxicity and muscle toxicity, improving overall organ function [112]. They help promote Müller cell proliferation and differentiation and stimulate Wnt-like pathways to improve retinal function in retinal regeneration [113]. In subcutaneous tumor models, ESC-exosomes exhibit anticancer properties and demonstrate antitumorigenic effects by inducing apoptosis, inhibiting growth, and promoting cancer cell reprogramming [114]. In aging and wound healing, they exert antisenescence effects by promoting angiogenesis, suppressing cellular senescence, and increasing wound healing in aging models [114,115]. Table 4 shows the potential of ESC-exosomes as a versatile and powerful regenerative medicine tool, which can intervene in several pathological processes in different systems. Functions in modulating inflammatory responses, promoting cellular repair and regeneration make them attractive candidates for therapeutic development.

**Table 4:** Therapeutic applications of ESC-derived exosomes in various animal models

**Disease targeted**	**Exosome source**	**Exosome size** **(average diameter)**	**Animal model**	**Treatment outcome**	**Reference**
Heart	mESCs	~39.7 nm	Mouse myocardial infarction	↑ Cardiac function ↓ Infarct size ↑ Neovascularization ↑ Progenitor cell survival	Khan *et al*. [116]
Heart	hESCs	50–125 nm	Mouse heart failure induced by transverse aortic constriction (TAC)	↑ Cardiac function ↑ Myocardial angiogenesis ↑ Microvessel integrity	Pang *et al*. [107]
Heart	mESCs	N/A	Mouse doxorubicin-induced cardiotoxicity (DIC)	↓ Inflammasome and pyroptosis ↑ M2 macrophages and IL-10 ↑ Cardiac function	Singla *et al*. [112]
Liver	hESCs	120–140 nm	Mouse liver fibrosis induced by carbon tetrachloride (CCl4)	↓ Liver fibrosis ↑ Liver regeneration and function	Wang *et al*. [108]
Lung	hESCs	~122.7 nm	Bleomycin (BLM)-induced pulmonary fibrosis mice model	↓ Lung inflammation and fibrosis ↑ Pulmonary function	Liu *et al*. [109]
Nerve	hESC-neurons	N/A	*in vitro* oxygen–glucose deprivation (OGD) model	↑Neuronal survival ↓ Inflammation and apoptosis ↑ Synaptic function	Deng *et al*. [110]
Bone	hESC-MSCs	78 nm	Mouse osteoarthritis with Destabilization of Medial Meniscus (DMM) surgery	↑ Cartilage protection ↑ Collagen II	Wang *et al*. [117]
Bone	hESCs	164.5 ± 63.7 nm	Rat ovariectomized (OVX) rat model with bone defect	↑ Osteogenesis ↑ Bone regeneration	Huang *et al*. [111]
Spine	hESCs	N/A	Rat Intervertebral Disc Degeneration (IVDD)	↓ Inflammasome and pyroptosis ↓ Intervertebral disk degeneration	Yu *et al*. [118]
Eye	hESCs	~140 nm	RCS rats with retinal degeneration	↑ Retinal cell proliferation ↑ Retinal function	Gao *et al*. [113]
Muscle	mESCs	N/A	Mouse doxorubicin-induced muscle toxicity (DIMT)	↓ Inflammasome and pyroptosis ↑ M2 macrophages ↑ Muscle function ↓ Atrophy and fibrosis	Dessouki *et al*. [119]
Cancer	hESCs	101 ± 7 nm	Subcutaneous tumor model in NOD-SCID mice	↓ Tumor size ↑ Cancer cell reprogramming ↑ Apoptosis	Zhou *et al*. [114]
Aging	mESCs	N/A	Aged ICR mice for wound healing	↓ Cellular senescence ↑ Fibroblast proliferation ↑ Wound healing	Bae *et al*. [120]
Aging	hESCs	50–150 nm	D-galactose-induced aged mice for wound healing	↑ Ulcer healing ↑ Aged angiogenesis ↓ Endothelial senescence	Chen *et al*. [115]

N/A indicates “not available”, meaning the data was either not collected, not recorded, or unavailable at the time of reporting

#### Induced pluripotent stem cell–exosome animal application

The details of the therapeutic use of the iPSC-derived exosomes in different animal models are presented in Table 5. In cardiac uses, exosomes derived from various iPSCs including cardiomyocytes, endothelial cells, smooth muscle cells, and MSCs have shown a lot of therapeutic potential [100,121–126]. The experiments conducted on mice, swine, and rats with myocardial infarction and ischemia/reperfusion injury have revealed better cardiac function, decreased cardiomyocyte apoptosis, increased autophagy, and smaller infarction size. For example, hiPSC-cardiomyocyte exosomes increase autophagy in myocardial infarction (MI) mice, while hiPSC-MSC exosomes decrease cardiac injury in a rat model of severe acute pancreatitis (SAP)–induced myocardial injury. Human iPSC-mesenchymal stromal cell–derived exosomes have been shown to have hepatoprotective effects in liver disease models [127,128]. Research on rat and mouse models of hepatic ischemia/reperfusion (I/R) injury reveals enhanced hepatoprotective effects, thus suggesting the possibility of using these exosomes for the treatment of liver diseases. Kidney and limb ischemia models have also been treated with iPSC-MSC-derived exosomes [129–131]. These exosomes decrease kidney injury, inflammation, and apoptosis in mouse renal I/R injury models and enhance limb ischemia and angiogenesis in mouse limb ischemia models. Skin wound healing and regeneration are significantly improved by several sources of iPSC-derived exosomes [132–137]. Such exosomes are known to increase re-epithelialization, narrow the scar area, and increase the maturation of collagen in the healing of wounds. Also, they promote wound healing in diabetic ulcers and second-degree burn wounds in mice and rhesus macaques, thus proving the versatility of the substance in dermatological care. Nervous system disorders are reported to experience positive changes with the use of iPSC-derived exosomes [138–142]. These exosomes derived from iPSC neurons and other sources have been shown to boost neurogenesis, circuit formation, functional recovery, and nerve regeneration in conditions such as spinal cord injury, Alzheimer’s disease, ischemic stroke, and peripheral nerve injury. These findings indicate their possibility in neuroregenerative medicine. Eye and bone models also use iPSC-MSC-derived exosomes for their advantage [143–148]. These exosomes improve bone formation, neovascularization, and osteogenesis in the contexts of bone defects and osteonecrosis as well as corneal and retinal injury and degeneration. Also, ovarian and vascular models have proved to be useful in the application of iPSC-derived exosomes with satisfactory outcomes [149,150]. They maintain fertility in premature ovarian insufficiency models and enhance granulosa cell proliferation. They also improve vascular function and decrease the aging markers in vascular dysfunction models. In summary, Table 5 shows that iPSC-derived exosomes can be used to treat various diseases in different animals and proves the therapeutic application of these exosomes. This collection of work offers a solid basis for further study and possible use of exosome-based treatments in patients.

**Table 5:** Therapeutic applications of iPSC-derived exosomes in various animal models

**Disease targeted**	**Exosome source**	**Exosome size** **(average diameter)**	**Animal model**	**Treatment outcome**	**Reference**
Heart	miPSCs	100 nm	Mouse myocardial ischemia/reperfusion	↓Cardiomyocyte apoptosis	Wang *et al*. [100]
Heart	hiPSC-cardiomyocytes	142 nm	Mouse myocardial infarction	↑Autophagy	Santoso *et al*. [122]
Heart	hiPSC- endothelial cells	100 nm	Mouse myocardial infarction	↑Cardiac function ↓Infarct size	Li *et al*. [125]
Heart	hiPSC-cardiomyocytes, hiPSC-endothelial cells, hiPSC-smooth muscle cells	98 nm	Swine myocardial infarction	↑Cardiac recovery	Gao *et al*. [121]
Heart	hiPSC-cardiomyocytes with normal or elevated cyclin D2 level expression	Normal cyclinD2-Exo (125 nm) or high cyclin D2-Exo (105 nm)	Swine myocardial infarction	↑Cardiac function ↓Infarct size	Zhao *et al*. [123]
Heart	hiPSC-MSCs	65 nm	Rat severe acute pancreatitis–induced myocardial injury	↑Cardiac function ↓Cardiac injury	Chen *et al*. [124]
Heart	hiPSC-cardiomyocyte	91 nm	Rat cardiac postsurgery model	↑Cardiac function ↓Surgical adhesions	Wang *et al*. [126]
Liver	hiPSC- mesenchymal stromal cells	135 nm	Mouse hepatic Ischemia/reperfusion injury	↑Hepatoprotection	Du *et al*. [128]
Liver	hiPSC- mesenchymal stromal cells	50–60 nm	Rat hepatic ischemia/reperfusion injury	↑Hepatoprotection	Nong *et al*. [127]
Kidney	hiPSC- MSCs	30–100 nm	Mouse renal ischemia/reperfusion injury	↓Renal injury ↓Inflammation ↓Apoptosis	Lim *et al*. [129]
Limb	hiPSC- MSCs	57 nm	Mouse limb ischemia	↓Limb ischemia ↑Angiogenesis	Hu *et al*. [130]
Limb	hiPSC-endothelial cells	95 nm	Mouse limb ischemia	↑Postnatal angiogenesis	Ye *et al*. [131]
Skin	hiPSCs	120 nm	Mouse wound healing in diabetic ulcer	↑Wound healing	Kobayashi *et al*. [133]
Skin	hiPSC- MSCs	30–100 nm	Rat wound healing	↑Re-epithelialization ↓Scar widths ↑Collagen maturity	Zhang *et al*. [132]
Skin	Rhesus macaque iPSCs (autologous vs. allogeneic)	100 nm	Rhesus macaque wound healing	↑Wound healing	Lu *et al*. [134]
Skin	iPSCs- keratinocytes	75 nm	Mouse deep second-degree abdominal burns	↑Wound healing ↑Angiogenesis ↑Re-epithelialization ↑Keratinocyte migration ↑Endothelial cell migration	Bo *et al*. [135]
Skin	Neural stem cells; hiPSCs	NSC-Exo (36–56 nm); hiPSC-Exo (50–105 nm)	Mouse skin trauma	↑Wound healing	Li *et al*. [136]
Skin	IFN-γ-primed iPSC-MSCs	114 nm	Mouse atopic dermatitis induced by *Aspergillus fumigatus*	↓Atopic dermatitis ↑Skin barrier ↓Inflammation	Yoon *et al*. [137]
Nerve	hiPSC-neurons	55 nm	Mouse dentate gyrus	↑Neurogenesis ↑Circuit assembly	Sharma *et al*. [138]
Nerve	Alzheimer’s patient–derived iPSC neurons	160 nm	Mouse Alzheimer’s pathology	↑Tau deposits ↑Alzheimer’s neuropathology	Podvin *et al*. [139]
Nerve	hiPSCs	142 nm	Rat peripheral sciatic nerve injury	↑Functional recovery ↑Nerve regeneration	Pan *et al*. [140]
Nerve	miPSCs	100 nm	Mouse spinal cord Injury	↑Motor function ↑M2 macrophage polarization	Li *et al*. [141]
Nerve	hiPSC-MSCs	70 nm	Mouse ischemic stroke	↑Neuroprotection ↑Functional recovery ↓Infarct size	Lu *et al*. [142]
Bone	hiPSC-MSCs	68.7 nm	Rat engineered tissue	↑Bone regeneration	Zhang *et al*. [143]
Bone	hiPSC-MSCs	83.3 nm	Rat bone defects	↑Angiogenesis ↑Osteogenesis	Qi *et al*. [144]
Bone	hiPSC-MSCs	100 nm	Rat steroid-induced osteonecrosis of the femoral head (ONFH)	↓Osteonecrosis ↑Angiogenesis	Liu *et al*. [145]
Bone	hiPSC-MSCs	110 nm	Mouse collagenase-induced osteoarthritis (OA)	↓Osteoarthritis	Zhu *et al*. [146]
Eye	hiPSCs	100 nm	Rat corneal epithelial defect	↑Corneal healing	Wang *et al*. [147]
Eye	hiPSC-retinal organoids	110 nm	Rat retinal degeneration	↑Retinal function ↑Structure recovery	Han *et al*. [148]
Ovarian	hiPSC-MSCs	124 nm	Cyclophosphamide-induced premature ovarian insufficiency -like mouse model	↑Granulosa cell proliferation ↓Apoptosis ↑Preserved fertility	Zhang *et al*. [149]
Vessel	miPSCs	148 nm	Aging-related vascular dysfunction in naturally aged mice	↑Angiogenesis ↑Vascular function ↓Aging markers	Li *et al*. [150]

#### Induced pluripotent stem cell–exosome clinical application

iPSC-generated exosome therapies are now being tested in various clinical studies for many medical conditions. In the refractory focal epilepsy for which iPSC-derived exosomes will be tried out intranasally in the form of drops, Peking Union Medical College Hospital in China (NCT05886205) will concentrate on overcoming the biological barrier and on the low immunogenicity. Likewise, a randomized, placebo-controlled trial (NCT06138210) is ongoing at Xuanwu Hospital in China to determine the effectiveness of intravenous iPSC-derived exosomes for acute ischemic stroke, the short-term condition. In another clinical trial at the Second Affiliated Hospital School of Medicine, Zhejiang University (NCT05738629) in China, its exosome drops for dry eye disease after refractive surgery are marked by the emphasis on their ocular surface healing ability. An early phase 1 trial (NCT05969717) at Peking Union Medical College Hospital in China is investigating the efficacy of iPSC-exosomes in managing atopic dermatitis, largely due to their anti-inflammatory and tissue repair properties. In France, Assistance Publique – Hôpitaux de Paris already launched a trial (NCT05774509) on the safety and effectiveness of EVs from iPSC-derived cardiovascular progenitor cells for treating nonischemic cardiomyopathies, expecting to recover cardiac function through repeated intravenous injections. These studies highlight the diverse therapeutic potential of iPSC-exosomes across different disease states, capitalizing on their natural capabilities for targeted, regenerative treatments. Here is the summary of these studies in Table 6.

**Table 6:** Overview of clinical trials on iPSC-derived exosome treatments

**NCT number**	**Condition**	**Intervention/Treatment**	**Study start ~ primary completion (Est.)**	**Enrollment (Est.)**	**Phase/Study type**	**Location**	**Injection route**	**Dosage**	**Primary outcome**	**Secondary outcome**
NCT05886205	Refractory focal epilepsy	iPSC-Exos (GD-iEXo-002)	2023-06-05 ~ 2025-06-13	34	Early Phase 1/Interventional	Peking Union Medical College Hospital, Beijing, China	Nasal drops	Four dosage groups as follows: 2 μg, 6 μg, 18 μg, or unspecified dose of iPSC-Exos in 200 μl, administered twice daily (bid) via nasal drip for 12 weeks.	Adverse events as assessed by CTCAE	Number of participants with abnormal vital signs and neurological examination, laboratory test results, and urine analysis
NCT05738629	Dry eye disease	PSC-MSC-Exo Eye Drops	2023–03 ~ 2025–02	12	Phase 1/Phase 2/Interventional	Second Affiliated Hospital School of Medicine, Zhejiang University, Hanzhou, Zhejiang, China	Eye drops	0.125 ml/single eye/one time, four times a day for 12 weeks	Improvement in Ocular Surface Disease Index (OSDI)	Tear secretion test, tear film break-up time, ocular surface staining score, Best Corrected Visual Acuity (BCVA), conjunctiva redness score, tear meniscus height, corneal confocal microscopy, and meibomian gland expressibility scores
NCT06138210	Acute ischemic stroke	hiPSC-Exos (GD-iExo-003)	2024-01-30 ~ 2025-08-30	29	Phase 1/Interventional	Xuanwu Hospital Beijing, China	Intravenous injection	Three dosage groups as follows: 20 μg/kg, 40 μg/kg, or 80 μg/kg of iPSC-Exos, administered once daily for 7 days	Number of participants who experienced dose-limiting toxicities (DLTs), safety, and preliminary efficacy in neurological recovery	Incidence of severe adverse events, favorable functional outcome, functional outcome, and changes in NIHSS score
NCT05969717	Atopic dermatitis	iPSC-Exos (GD-iExo-001)	2023-04-12 ~ 2025-04-30	20	Early Phase 1/Interventional	Peking Union Medical College Hospital, Beijing, China	Intravenous injection	Two dosage groups as follows: 10 μg/ml 50 μg/ml of iPSC-Exos, one drop (~50 μl) of iPSC-Exos was given to the affected skin area of 2–4 cm^2^ for 14 days, twice per day.	Adverse events as assessed by CTCAE	The proportion of subjects whose IGA and Eczema Area and Severity Index (EASI) score improved by 2 points or more compared with the baseline score
NCT05774509	Heart failure with reduced ejection fraction	Extracellular vesicle-enriched secretome of cardiovascular progenitor cells differentiated from induced pluripotent stem cells	2023-05-31 ~ 2025-08-15	12	Phase 1/Interventional	Assistance Publique - Hôpitaux de Paris, Paris, France	Intravenous injection	Two dosage groups as follows: 20x10E9 particles/kg or 40 × 10E9 particles/kg for three times	Serious adverse events and improvement in left ventricular function and symptoms	Validation of the bioactivity of the EV-enriched secretome by proliferation of human vascular endothelial cells, improvement in NYHA functional class, quality of life, and MACE

### Methods for engineering extracellular vesicles

**Figure 3:**
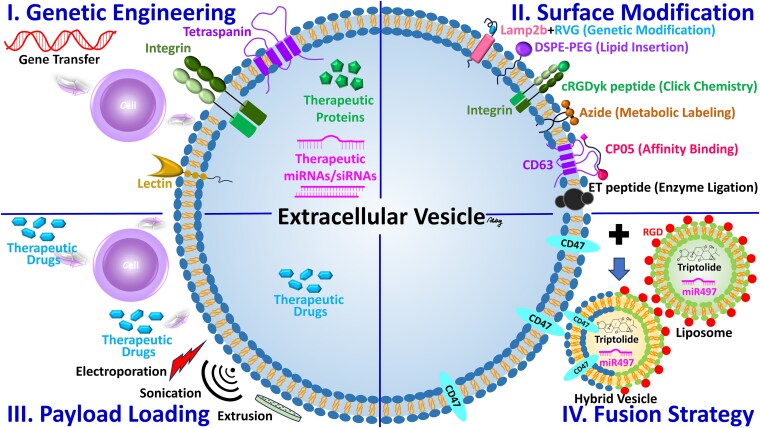
Extracellular vesicle engineering strategies for therapeutic applications. This schematic illustrates four primary strategies for engineering EVs to enhance their therapeutic potential. (i) ‘Genetic engineering’: Genetic modification of donor cells to express therapeutic genes and proteins. Techniques include gene transfer to incorporate specific genes encoding for therapeutic proteins, miRNAs, and siRNAs. These molecules are then packaged into EVs for delivery. II. ‘Surface modification’: Various methods to modify the surface of EVs for targeted delivery and improved functionality. This includes genetic modification of surface proteins (e.g. Lamp2b), lipid insertion (e.g. DSPE-PEG), click chemistry for peptide conjugation (e.g. cRGDyK binding to integrins), metabolic labeling with azide fed with azide-modified sugar, affinity binding of CP05 interacting with CD63, and enzyme ligation with ET peptide. (iii) ‘Payload loading’: Techniques to load therapeutic payloads into EVs, including active loading such as electroporation, sonication, and extrusion. Another method is passive loading, where cargo naturally incorporates into EVs during biogenesis. Manipulating the parent cell environment allows therapeutic small molecules or miRNAs to be loaded. For example, incubating donor cells with paclitaxel results in EVs, particularly MVs, carrying the drug as they inherit the donor cell contents. This strategy enables the encapsulation of therapeutic drugs, proteins, and nucleic acids within EVs for delivery to target cells. IV. ‘Fusion strategy’: This involves creating hybrid vesicles by fusing EVs with other nanocarriers, such as liposomes. Liposomes can encapsulate therapeutic agents, such as triptolide and miR497. CD47 can help evade the mononuclear phagocyte system. This strategy enhances the loading capacity and stability of therapeutic agents. Hybrid vesicles can carry combined therapeutic agents and CD47 for synergistic effects. The integration of these engineering strategies enhances the therapeutic efficacy and targeting capability of EVs, making them versatile tools for biomedical applications

Techniques in bioengineering have been identified to be useful in controlling the content and functions of EVs and they include genetic engineering, surface modification, payload loading, and fusion strategy [151–153]. These approaches allow for the manipulation of EVs’ content and functionality, thus opening the door to novel applications in diagnostics, drug delivery, and tissue repair. A detailed illustration of these methods can be found in Figure 3.

#### Genetic engineering

In the context of bioengineering, one of the most effective approaches is to genetically manipulate the parent cells to change the payload and characteristics of EVs. Due to the precise control over the cargo of EVs in regard to regenerative medicine, drug delivery, and diagnostics, this therapeutic potential is significantly improved. Thus, genetic engineering methods can be used to enhance EV synthesis, their stability, and the level of their purity. Genetically modified EVs derived from donor cells are considered more effective as drug delivery systems. This process involves the introduction of the donor cells with the aim of having them secrete some proteins, nucleic acids, or drug molecules. Consequently, bioactive cargos of interest are incorporated into EVs, which are then derived from the donor cell culture supernatant. Genetic modification, widely utilized in different biomedical disciplines, gives an opportunity to control the gene activity in cells. In terms of changing gene expression, researchers have delivered both native and non-native functional oligonucleotides into parent cells; thus, the cellular products including DNA, RNA, lipids, and proteins are encapsulated in the membrane or lumen of EVs.

This approach involves the derivation of parental cells, which are involved in the formation of EVs, and the optimization of EVs for the production of secreted therapeutic proteins. These cells can be genetically or metabolically modified in a way that the expression of surface markers of the EVs is affected, and this, in turn, improves the targeting ability and biocompatibility of the EVs. This is done by incorporating the coding region of the ligand of interest between the signal peptide and the N-terminus of the mature part of a transmembrane protein. The gene transfer is carried out with the help of a retrovirus or lentivirus vector, which enables the package to pass on and get expressed in the parental cells. Therefore, the parental cells transfected with the desired peptide produce EVs with the peptide on their surface or the engineered EVs that secrete therapeutic proteins.

(1) An instance of genetic engineering concerning EVs is the modification of parent cells to produce higher amounts of therapeutic proteins or regulatory molecules that are packaged in the resultant vesicles. For instance, cells engineered from parents to produce more growth factors such as VEGF or insulin-like growth factor (IGF) will secrete EVs that are loaded with these potent factors and can stimulate new blood vessel formation and tissue repair when delivered to the body. Xiao *et al*. designed EVs for central nervous system (CNS) delivery by transducing neural stem cells with the fragment of platelet-derived growth factor A (PDGFA) through lentivirus [154]. The primary oligodendrocyte progenitor cells *in vitro* were observed to have a higher binding efficiency to these engineered EVs, thus improving their targeting ability.

The proteins located on the surface of exosomes include tetraspanins, integrins, and lectins that play roles in determining the targeting of the exosomes to the target receptors of the cells. This is achievable by changing the expression of certain proteins in the parent cells that are to be delivered by the exosomes, and this can greatly improve the targeting efficiency of the engineered exosomes. Tetraspanins are a family of proteins that play a role in vesicle-cell fusion as well as signaling. For instance, the CD63-positive exosomes have a preference for the cells with Tspan8 while the downregulation of Tspan8 in mice leads to reduced exosome uptake in the liver and increased accumulation in the spleen, brain, and heart [155,156]. Manipulating the levels of tetraspanin can affect the biodistribution of exosomes since tetraspanins also interact with tissue-specific lectins, which help in the adhesion of exosomes to dendritic cells via the DC-SIGN receptor as well as exosomal CD81 to the hepatocyte asialoglycoprotein receptor via galectin domains [157]. It can hence be concluded that overexpression of tetraspanins can improve targeting. Galectins are β-galactoside-binding proteins that mediate glycans’ roles in cell and vesicle interactions. For example, galectin-9 overexpression triggers the shedding of galectin-9 positive exosomes from tumor cells and supports angiogenesis [158].

(2) It involves engineering of the parent cells to secrete miRNA of interest, thus ensuring that the miRNA is incorporated into the EVs for therapeutic application. Designed EVs with miRNA having anti-inflammatory or antitumor effects can affect the target cells and the processes of diseases. Due to the high specificity of genetic engineering, it is possible to load EVs with certain cargo that is targeted to certain cellular pathways or functions in order to increase the therapeutic effect and individual approach. New developments in genetic engineering may enhance and extend the function of EVs in numerous clinical applications, thus providing useful resources for state-of-the-art, specific therapies. Alvarez-Erviti *et al*. showed that siRNAs can be delivered into the mouse brain using exosomes that are prepared from dendritic cells and genetically modified to express neuron-specific rabies viral glycoprotein (RVG) peptide [159]. These exosomes were able to transport siRNA into brain cells and, therefore, led to efficient gene silencing, which proves the possibility of RNA-based therapeutics in diseases such as Alzheimer’s. Ohno *et al*. expressed that exosomes carrying anti-tumor miRNA against epidermal growth factor receptor (EGFR) can be used to treat breast cancer [160]. Thus, the researchers here genetically modified donor cells to secret exosomes containing let-7a miRNA through the expression of a specific peptide and these exosomes selectively targeted let-7a miRNA to EGFR-positive cancer cells in mice, suggesting the possibility of cancer treatment through exosome. Kim *et al*. showed that CRISPR/Cas9-loaded exosomes could promote apoptosis of ovarian cancer cells through downregulation of poly (ADP-ribose) polymerase-1, which indicates the potential of gene editing exosomes in anti-cancer treatment [161]. Another study revealed that M1 macrophage-derived exosomes modified to bind IL-4 receptor prevented tumor development by converting tumor-associated macrophages (TAMs) to M1-like macrophages [162]. These exosomes, transfected with NF-κB p50 siRNA, miR-511-3p, and IL4RPep-1, were efficiently internalized into M2 macrophages, suppressed the expression of their target genes, reduced M2 markers, and enhanced M1 markers. Systemic administration retarded the tumor growth, modulated cytokine production, and boosted antitumor immunity, thus providing a potential cancer immunotherapy.

#### Surface modification

Surface modification is one of the key aspects of the engineering approaches used in the context of EVs since it provides a versatile set of tools for regulating the contact between the vehicles and their target cells and tissues. This technique entails the controlled manipulation of lipid and protein contents of EV membranes with the aim of improving their stability, targeting efficacy, and overall efficiency. An example of such a strategy is the conjugation of targeting moieties to the surface of EVs. Surface modification also increases their specificity and effectiveness when used in the body and decreases the adverse effects. Many strategies have been employed for EV surface engineering such as genetic engineering of cells, lipid insertion, click chemistry, metabolic labeling, affinity binding, and enzymatic ligation. These methods collectively enhance the therapeutic efficacy of EVs by enhancing the targeted and effective delivery of the therapeutic molecules to the targeted cells or tissues.

##### Genetic modification

This entails modifying donor cells to display targeting groups on the surface of the EVs. Targeting peptides or proteins are joined to the vesicle membrane proteins or lipid-binding proteins/peptides. Thus, the targeting elements end up being presented on the surface of the resulting EVs in order to improve their ability to recognize certain cells or tissues. For instance, Lamp2b-RVG is one of them. The Lamp2b protein, which is an exosomal membrane protein, has the targeting brain peptide RVG, RVG genetically incorporated into it. This modification enables EVs to selectively transport BACE1 siRNA into neurons for Alzheimer’s disease treatment [159] or HMGB1 siRNA for ischemic stroke treatment [163].

##### Lipid insertion

The targeting molecules conjugated with lipid fragments are loaded into the EV membrane using simple and standard mixing and incubation methods. This method is rather simple and does not affect the structure and biological characteristics of EVs, which is why it is widely used for surface modification. For instance, in the case of DSPE-PEG-RGD, the RGD peptide that has high specificity toward the integrin on the blood vessels is attached to the DSPE-PEG lipid and incorporated onto the EV surface. This method has been applied to increase the efficiency of EVs to target blood vessels and thus to induce therapeutic angiogenesis and to improve the imaging of angiogenesis [164]. Another conjugation method, DSPE-PEG-AA, has been employed to increase the binding of EVs to tumor vasculature, thus increasing the delivery of drugs like paclitaxel (PTX) [165].

##### Click chemistry

This type of chemical ligation entails the use of bioorthogonal reactions that can label targeting groups to the surface of EVs without affecting the normal functions of the EVs. It enables specific and effective alteration of EV surfaces with different bioactive molecules. The cyclic RGDyK peptide, which has specificity for integrins, is attached to the surface of EVs through azide-alkyne cycloaddition. This chemical ligation strategy is applied for the engineering of EVs for the targeting of ischemic brain tissue in the transient middle cerebral artery occlusion mice model to deliver therapeutic molecules such as curcumin to effectively minimize inflammation and cell death in the affected area [166].

##### Metabolic labeling

The metabolic substrates that are used to feed the cells are altered in some way, for instance, azide-modified sugars that are incorporated into the EV biogenesis process, thus producing EVs that have surface molecules that can be further altered. This method offers a strategy to functionalize the EV surface for targeted delivery applications. For instance, Wang *et al*. treated the EV-secreting cells with *L*-azidohomoalanine or tetra-acetylated *N*-azidoacetyl-*D*-mannosamine for generating azide-containing EV [167]. These EVs could be conjugated with fluorescent dyes or biotin through click chemistry and then be employed for imaging as well as drug delivery.

##### Affinity binding

This modification entails the surface targeting moieties on the EV surface through conjugation with the affinity molecules of the EV membrane proteins or lipids. This method is rather weaker than covalent bonding; however, it does not affect the integrity of the EV membrane. Based on this technology, the targeting moieties that can be used are peptides, proteins, or aptamers and the modification is done through simple mixing and incubation. For instance, CD63 is a biomarker for exosomes; therefore, the affinity peptide from CD63, CP05, can effectively coat the surface of the exosomes. This was illustrated in the use of CP05-modified exosomes with muscle-targeting peptide M12 in mdx mice for treating Duchenne muscular dystrophy and the drug phosphorodiamidate morpholino oligomer (PMO). This approach increased the number of myofibers with dystrophin without observable toxicity [168]. Aptamers are a relatively mild and specific tool for binding with the target. For example, a method was proposed for the surface functionalization of exosomes with DNA aptamers and DNA hybridization chain reaction, which enables the selective conjugation of FITC, which can be replaced by other target molecules [169].

#### Enzymatic ligation

Protein ligase, for instance, is utilized to covalently incorporate targeting molecules on the surface of EVs. This method can be very selective and can yield good results in terms of altering the EVs for specific therapeutic applications. Pham *et al*. employed Sortase A and OaAEP1 ligases to bioconjugate the target peptide to red blood cell–derived EVs (RBCEVs) at ~380 peptide copies per vesicle [170]. They also developed the EGFR-targeting ET peptide and improved the delivery of paclitaxel to EGFR-positive lung cancer cells and thus improved the treatment outcomes in a mouse model of EGFR-positive lung cancer. Thus, the targeting moieties are conjugated to the EV membrane through covalent linkages, which increases the stability and selectivity of targeting for therapeutic purposes.

#### Payload loading techniques

Payload loading techniques are very crucial in the development of EVs as they enable the engineers to design these vesicles to fit a certain therapeutic application by loading them with different biomolecules including small molecules, nucleic acids, and proteins.

(1) The first mechanism is passive loading, which is a distinguishable strategy where cargo is somehow naturally packaged into EVs during their formation. For instance, if the parent cell environment is manipulated, then therapeutic small molecules or endogenous molecules like miRNA can be encapsulated into EVs that would then affect the packaging mechanisms that occur during the formation of ILVs within MVBs. Thus, when donor cells are treated with certain molecules, including internalized drugs, EVs, particularly MVs, acquire these molecules as they contain the contents of the donor cells. Pascucci *et al*. were able to encapsulate PTX within mesenchymal stromal cells and obtained PTX-loaded exosomes, which showed high anti-proliferative activity against pancreatic cancer cells [171]. Nevertheless, this method is likely to have poor loading efficiency because it is not specific and can only be applicable to drugs that can easily diffuse across the plasma membrane.

Another passive method of loading EVs with cargo entails incubating EVs with the cargo at room temperature or 37°C, and the cargo is internalized into the EVs. This simple method can be used for small molecules, nucleic acids, proteins, and nanomaterials; the loading efficiency is influenced by the hydrophobicity of the cargo, which allows for an interaction with the lipid membrane [172,173]. It should be noted that, besides passive diffusion, some molecules can be internalized in EVs actively. For instance, Betzer *et al*. showed that glucose-coated gold nanoparticles could be internalized into EVs through an active process [174].

(2) Active loading methods are very precise and controlled when it comes to loading the cargo into the EVs. Some of the techniques include electroporation where an electric field is applied to the EVs and the cargo molecules, this creates an opening on the EV membrane to allow for the loading of therapeutic agents. This technique is highly useful for loading nucleic acids like small siRNAs or mRNAs to EVs to be used in gene therapies as first proposed by Alvarez-Erviti *et al*. [159]. siRNA was incorporated into the brain targeting exosomes to exert its functions in the brain. Another significant technique is sonication, in which the hydrophobic molecules like chemotherapeutic drugs get encapsulated by applying ultrasonic energy. Sonication disrupts the vesicle membrane thus facilitating the cargo entry and is ideal for loading drugs, proteins, peptides, and nanomaterials but not nucleic acids due to the risk of degradation [175,176]. Sonication is effective and relatively easy to perform, but it is not appropriate for large-scale production. Extrusion, in which vesicles and cargo are mixed and forced through a lipid extruder with pores of 100–140 nm in size, leads to high loading and vesicles of the same size, but the membrane structure is changed and cytotoxicity may occur [177,178]. Freeze–thaw cycling that compromises the vesicle membrane through a series of freeze–thaw cycles to enable the diffusion of the cargo is ideal for drug and protein loading; however, it may affect the protein activity, thus reducing potency [179].

#### Fusion strategy

The last approach is to develop the so-called hybrid EVs where EVs are combined with drug-loaded liposomes by fusion strategy. This approach uses lipid bilayer fusion which is a natural process in cell biology for the engineering of EV membrane. Afterward, when liposomes are incubated with EVs, such structures spontaneously fuse and the functional groups of the liposomes will be presented on the surface of the fused vesicles. Hence, Li *et al*. synthesized new hybrid nanoparticles named HENPs, in which RGD-liposomes were conjugated with CD47-bearing EVs for the transportation of triptolide (TP) and miR497 to cisplatin-resistant ovarian cancer cells [180]. The RGD modification provided tumor targeting and CD47 avoided the mononuclear phagocyte system (MPS). In the xenograft model with SKOV3-CDDP tumors, HENPs efficiently accumulated within tumors and prevented liver trapping by the MPS. Furthermore, the miR497/TP loaded HENPs exhibited a great antitumor property, which demonstrated that they could serve as a promising system for miRNA and small molecule drug delivery in cancer treatment.

#### Engineering strategies for improved extracellular vesicle targeting

An overview is given of engineering strategies for the improvement of EV targeting, as discussed in Table 7, in which genetic engineering, surface modification, payload loading techniques, and fusion strategies are all capable of leading to a paradigm shift in EV research. These approaches improve EV targeting by manipulating surface markers, incorporating functional cargo, and combining EVs with synthetic carriers. Genetic engineering tailors parent cells to modify EV composition and receptor-specific interactions, such as tetraspanins for biodistribution and ligand encoding for tissue-specific delivery. Surface modification methods, including ligand conjugation and metabolic labeling, enhance the precision and efficacy of EV interaction with target tissues. Payload loading techniques, both passive and active, ensure the encapsulation of therapeutic molecules, aligning cargo with specific disease pathways to achieve superior targeting. Advance targeting and functional delivery are achieved by leveraging the natural properties of both EVs and synthetic vesicles, forming hybrid systems. These together help form a robust framework for further development of EV-based diagnostics and therapies.

**Table 7:** Engineering strategies for improved extracellular vesicle targeting

**Strategy**	**Principle**	**Key technique**	**Mechanism of targeting enhancement**	**Example**
**Genetic engineering**	Genetic engineering allows precise control over the properties of parental cells to modify EV cargo and surface markers, improving targeting efficiency	Surface protein engineering	By altering the expression of surface proteins such as tetraspanins (CD63, CD81), integrins, and lectins, EVs can be tailored to interact with specific receptors on target cells	● CD63-positive exosomes show a preference for Tspan8-expressing cells. Manipulating Tspan8 expression in parent cells affects exosome biodistribution, enhancing accumulation in specific tissues such as the spleen, brain, and heart ● Tetraspanins interact with tissue-specific lectins, such as DC-SIGN in dendritic cells or galectin domains in hepatocytes, enabling precise targeting
			Galectin overexpression can enhance EV adhesion to vascular or tumor environments, improving delivery to angiogenic sites	● Galectin-9 overexpression on EVs for tumor targeting
		Genetic delivery of targeting ligands	Engineering parent cells to express targeting peptides fused to transmembrane proteins like Lamp2b enables EVs to specifically bind to receptors on target cells	● Lamp2b-RVG targets neurons for treating neurological diseases such as Alzheimer’s ● Modified EVs expressing tumor-targeting peptides deliver miRNA or siRNA to cancer cells, enhancing antitumor efficacy
		Cargo optimization for functional targeting	By encoding miRNA, siRNA, or therapeutic proteins in parent cells, EVs can carry specific functional cargo that regulates disease pathways	● siRNA-loaded EVs targeting EGFR have been used to silence genes in breast cancer cells, demonstrating precise targeting and therapeutic outcomes
**Surface modification**	Surface modification enhances EV targeting by directly manipulating the lipid or protein composition of the EV membrane to improve interaction with target tissues	Ligand conjugation	EVs can be functionalized with ligands, peptides, or antibodies to enhance binding to specific cellular receptors	● DSPE-PEG-RGD: Incorporating RGD peptides targets integrins on endothelial cells, improving angiogenesis therapy and tumor imaging ● CD63-CP05 Peptide: Affinity binding to CD63 enables muscle-specific targeting in Duchenne muscular dystrophy
		Click chemistry	Surface bioorthogonal reactions attach functional groups such as RGD peptides to EVs, improving their specificity for ischemic brain tissues in stroke models and this enables EVs to deliver therapeutic molecules like curcumin directly to injured tissues	● RGDyK-modified EVs target ischemic brain tissue to deliver curcumin, reducing inflammation and cell death
		Metabolic labeling	Using azide-modified sugars during EV biogenesis allows subsequent functionalization with targeting molecules through click chemistry, and this technique enables fluorescent labeling or drug delivery for imaging and therapy	● Azide-labeled EVs generated for targeted functionalization and therapeutic applications
**Payload loading techniques**	Payload loading focuses on encapsulating therapeutic cargo within EVs, which can improve targeting by aligning the cargo with specific disease pathways or cellular mechanisms	Passive loading	Altering the cellular environment during EV biogenesis ensures selective incorporation of therapeutic molecules	● Treating MSCs with paclitaxel generates PTX-loaded EVs, which preferentially target and inhibit cancer cells
			Incubating EVs with hydrophobic drugs or siRNA enables their passive encapsulation and targeted delivery	● BACE1 siRNA-loaded exosomes targeting neuronal pathways
		Active loading	Techniques like electroporation or sonication create temporary pores in the EV membrane, allowing precise loading of therapeutic molecules such as siRNA, mRNA, or proteins	● Electroporated EVs carrying siRNA targeting neuronal pathways have shown success in silencing genes in the brain, improving targeting for neurological diseases ● Sonicated EVs encapsulating chemotherapeutic drugs enhance drug delivery to tumor cells, reducing off-target effects
**Fusion strategy**	The fusion strategy combines EVs with synthetic vesicles or liposomes to create hybrid systems that enhance targeting and functionality	Hybrid vesicles	Fusion of EVs with RGD-functionalized liposomes creates hybrid vesicles capable of targeting integrins on tumor vasculature, delivering miRNA and chemotherapeutics to resistant cancer cells	● RGD-liposomes fused with EVs for tumor targeting
			Incorporating EVs with CD47-bearing liposomes allows for immune evasion, enabling the hybrid vesicles to bypass the mononuclear phagocyte system and accumulate at tumor sites	● CD47-EV hybrids for immune evasion
		Enhanced targeting efficiency	By leveraging the natural targeting properties of EVs and the customizable features of liposomes, hybrid vesicles achieve precise delivery of therapeutic agents, such as triptolide (TP) or miR-497, in xenograft cancer models	● Hybrid EV-liposomes deliver triptolide or miR-497 for cancer therapy

#### Engineering strategies for improved stability of extracellular vesicle

To achieve this, the stability and half-life of engineered EVs *in vivo* are critical to maintaining their integrity and function in biological settings for extended duration. These properties are enhanced through surface modification, genetic engineering, loading of payload, and the development of fusion strategies to engineer EVs. Factors influencing stability and half-life primarily include membrane composition, immune system interaction, enzymatic degradation, and size and surface charge.

(1) Membrane composition: The stability of EVs is because they contain the lipid bilayer structure enriched with cholesterol, sphingomyelin, and phospholipids. However, PEGylation (polyethylene glycol coating) and/or incorporation of biomimetic molecules increase resistance to enzymatic degradation and immune clearance, prolonging half-life [181,182].

(2) Immune system interaction: Due to the fact that EVs may be identified by the immune system, they may be phagocytosed by macrophages or opsonized for clearance via the MPS. Reducing immune recognition and extending circulation time can be achieved through strategies of surface engineering such as cloaking EVs with CD47 (“don’t eat me” signal) or other immune evasive molecules.

(3) Enzymatic degradation: EV membranes may be also subjected to enzymatic degradation in the bloodstream and other biological environments. Enriching membrane rigidity with cholesterol or incorporating cross-linked lipids can increase resistance against degradation [153,183].

(4) Size and surface charge: EVs of smaller size, such as exosomes, usually have longer circulation times than larger EVs, such as microvesicles or apoptotic bodies. Additionally, neutral or slightly negative surface charges help evade nonspecific uptake and promote longer systemic circulation [184,185].

A summary of the strategies for improving the stability and half-life of engineered EVs, and their importance in enhancing therapeutic efficacy and *in vivo* performance, are presented in Table 8.

**Table 8:** Engineered strategies to improve stability and half-life of extracellular vesicles

**Engineering strategy**	**Key technique**	**Mechanism of stability enhancement**	**Example**
**Genetic engineering**	- Enhanced membrane composition - Surface protein expression - Therapeutic cargo stabilization	- Optimizes lipid and protein composition for resistance to enzymatic degradation - Improves EV integrity with structural proteins - Ensures functional encapsulated cargo during circulation	- Increased tetraspanin expression for enhanced membrane stability - miRNA incorporation to resist nucleases
**Surface modification**	- PEGylation - Lipid insertion - Bioorthogonal labeling	- Reduces immunogenicity and prevents opsonization - Enhances structural integrity of EV membranes - Maintains stability while adding targeting ligands	- DSPE-PEG-RGD for improved stability and targeting - Click chemistry for ligand attachment without structural alteration
**Payload loading techniques**	- Active loading (e.g. electroporation, sonication) - Passive loading	- Protects therapeutic molecules by maintaining EV membrane integrity - Minimizes damage during biogenesis and encapsulation	- Electroporation for siRNA loading - Passive loading for encapsulating small molecules
**Fusion strategy**	- Hybrid vesicle formation - Immune evasion	- Combines natural EV stability with synthetic lipid robustness - Avoids phagocytosis by mononuclear phagocyte system	- RGD-liposome and CD47-bearing EV fusion (HENPs) for enhanced stability and tumor targeting

In using engineering strategies to increase EV stability, the following must be considered:

(1) Rapid clearance by the liver and spleen: A large percentage of systemically injected EVs are quickly cleared by the liver and spleen, limiting their bioavailability [186–188]. Possible future strategies might involve the use of receptor-specific blocking or decoy molecules to redirect EVs to paths not producing clearance.

(2) Interaction with the immune system: Immune responses and/or premature clearance by opsonization may occur with unmodified EVs. Promising solutions are to cloak the EVs with PEG or biomimetic coatings.

(3) Designing long-circulating EVs: Hybrid EV-liposome technologies or the use of synthetic material integrated into EVs may provide new means of prolonging circulation time without losing functional properties.

(4) Quantifying stability and half-life: To compare engineered modifications, standardized methods to measure EV half-life and stability *in vivo* are needed. The biodistribution and degradation rate of EVs can be gained with techniques like radiolabeling or fluorescent tracking.

For clinical utility in drug delivery, gene therapy, and regenerative medicine, the stability and half-life of EVs is critical. Extending circulation time can increase EV accumulation in target tissues and increase therapeutic efficacy while decreasing the amount of EVs required. As an additional benefit of improved stability, off-target effects and systemic toxicity are minimized, which are both essential for clinical translation. The stability and half-life of engineered EVs *in vivo* are enhanced through a combination of genetic engineering, surface modification, payload loading, and fusion strategies. To overcome issues of enzymatic degradation, immune clearance, and short circulation times, these approaches entail tailoring membrane composition, surface properties, and methods of cargo encapsulation.

### Engineered embryonic stem cell–vesicle animal application

#### Cancer therapy

The potential for therapeutic applications of ESC-derived exosomes, specifically in cancer and neural restoration has been shown in engineered strategies (Table 9) [189–193]. In cancer therapy and vaccines, Kavitha Yaddanapudi and Shuhan Meng group demonstrated that ESC-derived exosomes genetically engineered to overexpress granulocyte-macrophage colony-stimulating factor (GM-CSF) function as cell-free vesicles to boost immune responses against tumors [189–191]. For example, prophylactic cancer vaccines with increased GM-CSF were genetically engineered and significantly reduced tumor incidence and increased immune response in mouse models of lung cancer. It effectively blocked tumor metastasis to the lungs as well as enhanced antitumor immune cell activity while reducing immunosuppressive cells such as regulatory T cells. Furthermore, Zhu *et al*. revealed that PTX-loaded ESC-exosomes conjugated with peptides like c(RGDyK) could enhance the efficiency of drug delivery to glioblastoma, particularly resulting in enhanced tumor suppression and survival of xenograft mouse models [192]. These results suggest that Surface modifications of ESC-exosomes with tumor-targeting ligands or encapsulation of specific therapeutic molecules enhance their efficacy and targeting capabilities. Moreover, ESC-derived exosomes should provide a safer alternative to live stem cells and avoid the risk of teratoma formation and immune rejection.

**Table 9:** Applications of engineered vesicles derived from ESCs and their derivative cells in various animal models

**Disease targeted**	**Vesicle source**	**Exosome size** **(average diameter)**	**Engineered vesicle method**	**Animal model**	**Treatment outcome**	**Reference**
Cancer	GM-CSF expressing mESCs (ES-D3)	30–100 nm	GM-CSF overexpression via genetic engineering (lentiviral transduction)	Mice with Lewis lung carcinoma (LLC)	Effective prophylactic cancer vaccine, significant reduction in tumor incidence and enhanced immune response	Yaddanapudi *et al*. [189]
Cancer	GM-CSF expressing mESCs (ES-D3)	30–100 nm	GM-CSF overexpression via genetic engineering (lentiviral transduction)	N/A	Characterization of GM-CSF expressing mESCs (ES-D3)	Meng *et al*. [190]
Cancer	GM-CSF expressing mESCs (ES-D3)	30–100 nm	GM-CSF overexpression via genetic engineering (lentiviral transduction)	Mouse metastatic lung cancer	Inhibited growth of metastatic lung tumors	Meng *et al*. [191]
Cancer	hESCs	ESC-Exos (70 ± 18 nm); Exo-PTX (107 ± 20 nm); cRGD-Exo-PTX (125.2 ± 27 nm)	cRGD-Exo-PTX: Exosomes conjugated with c(RGDyK) peptide and loaded with the drug PTX	Subcutaneous xenograft and orthotopic glioma bearing nude mouse model	Enhanced drug delivery to glioblastoma; significant tumor volume reduction and improved survival in animal models	Zhu *et al*. [192]
Nerve	mESCs	118 ± 49.8 nm	Curcumin loading via freeze–thaw cycles into ESC-derived exosomes	Mouse brain reperfusion/Injury	Restored neurovascular unit integrity; reduced inflammation, oxidative stress, and ischemic lesion volume	Kalani *et al*. [193]

N/A means “not applicable”, only in vitro experiments, no animal research included

#### Stroke therapy

In neuroprotection and restoration, Kalani *et al*. indicated that ESC-exosomes loaded with curcumin (anti-inflammatory and neuroprotective agent) using freeze–thaw cycles restored neurovascular unit integrity in mouse models of brain ischemia–reperfusion injury [193]. Therapy reduced ischemic lesion volume, inflammation, and oxidative stress, with the potential for stroke recovery. The exosome formulation successfully restored tight junction proteins in endothelial cells, contributing to neurovascular protection following ischemic damage​. These results indicate that ESC-exosomes are intrinsically targetable, free of toxicity, and able to transverse physiological barriers, such as the BBB. These properties make these carriers particularly advantageous as therapeutic carriers. Furthermore, this poses superior scalability due to the high proliferative potential of ESCs for clinical translation. Therefore, engineered ESC-derived exosomes exhibit versatility and efficacy in applications ranging from cancer immunotherapy and targeted drug delivery to neurovascular restoration. The potential of these innovations to be game-changing therapeutic approaches for many diseases is highlighted.

### Engineered induced pluripotent stem cell-vesicle animal application

#### Regenerative medicine

The use of engineered EVs in regenerative medicine, especially those from PSCs, is a revolutionary approach in biomedical science and therapeutics. Pluripotent stem cells are cells that have the potential to develop into many different cell types and are therefore considered a promising source for cell therapies. The EVs that originate from these PSCs retain this regenerative capacity and function as signaling molecules involved in tissue repair and regeneration. An example of the use of engineered EVs in regenerative medicine is in cardiac repair. The PSC-derived EVs have been found to be enriched with growth factors, miRNAs, and proteins that enhance cardiac function after an injury. For example, vesicles derived from iPSC-cardiomyocytes (iCMs) encapsulated in a hydrogel patch (3 × 10^10^ vesicles) were applied in a rat model of myocardial infarction [126]. These vesicles with a diameter of 70–100 nm had a considerable effect on the reduction of arrhythmic burden, improvement of ejection fraction, decrease of cardiomyocyte apoptosis, reduction of the infarct size, and prevention of cell hypertrophy. Consequently, engineered EVs generated from PSCs can potentially enhance the formation of new blood vessels, combat inflammation, and enhance the survival of cardiomyocytes. These regenerative effects can enhance cardiac performance and decrease the formation of scar tissue, making it a potential therapeutic strategy for cardiovascular diseases.

In the context of osteoarthritis or bone fractures, the application of EVs obtained from PSCs has been evidenced to regulate inflammation, promote chondrogenesis, and improve cartilage and bone tissue repair. The cargo of regenerative properties, which includes growth factors and extracellular matrix components, help in the rebuilding of the musculoskeletal system, and this can be a way of enhancing the results of orthopedic procedures. Hence, engineered EVs from iPSCs can be used as a non-invasive therapeutic strategy for tissue repair. For example, Gui *et al*. propose a new drug delivery system in the form of bone-targeted engineered exosomes (BT-Exo) to deliver siRNA directly to osteoblasts for the treatment of osteoporosis [194]. These exosomes originate from hiPSC-derived mesenchymal stem cells (iMSCs), and the surface of these exosomes is conjugated by a bone-targeting peptide, SDSSD. SiRNA targets the Shn3 gene that is central to bone resorption and formation in the body. This work shows that BT-Exo-siShn3 promotes osteogenesis and angiogenesis, suppresses osteoclastogenesis *in vitro*, and prevents bone loss *in vivo* through increasing type H vessel formation and decreasing bone resorption indices. This engineered exosome system can be considered as a highly effective and systematic solution for treating osteoporosis taking advantage of both iMSC-derived exosomes and siRNA therapy.

To improve corneal damage, exosomes from iPSC-MSCs were loaded into thermosensitive chitosan-based hydrogel [195]. Thus, the exosomes with miR-432-5p enhanced the process of healing, decreased scar formation, downregulated the collagen level and promoted the regeneration of epithelium and stroma in the rat model of corneal injury.

#### Drug delivery

The use of engineered EVs in drug delivery has presented a revolutionary approach as a drug delivery platform with immense possibilities for targeted and stimuli-responsive drug release. The engineered EVs such as those derived from PSCs offer an opportunity to overcome some of the challenges that are associated with conventional drug delivery systems. EVs can be designed to contain chemotherapeutic drugs, siRNA, or other therapeutic loads, and this makes them useful in targeted drug delivery to the target cells. Engineered EVs present a potential approach for the administration of nucleic acid–based therapeutics. Small RNAs like miRNA or siRNA can be incorporated into EVs so that the natural function of EVs in communication between cells can be used for gene regulation. For example, engineered EVs produced from iPSCs have revealed a high potential for various biomedical uses. Such EVs can be loaded with particular drugs like anti-inflammatory agents, antifibrotic substances, and procardiomyogenic factors, which can greatly improve the treatment outcome.

In one study, iPSC-derived monocyte extracellular vesicles (mEVs) were loaded with antagomir to miR-155, which is involved in inflammation [196]. The engineering process was to transfect the EVs with antisense miR-155 (antagomir-155), and the ExoQuick-TC was used to purify the EVs to ensure effective loading. These mEVs were engineered to selectively bind to the peripheral monocytes and thus reduced monocyte activation and inflammation. This means that engineered mEVs could be employed to manipulate immune responses and dampen inflammation in diseases like human immunodeficiency virus (HIV) infection where inflammation is a major problem. Another work describes the development of a bone-targeted engineered exosome platform for the delivery of Shn3 siRNA for the treatment of osteoporosis [194]. The engineering approach employed the use of a bone-targeting peptide to coat the surface of the exosome so that only bone tissues can be targeted. This targeted delivery system was able to decrease bone loss and stimulate bone formation in animal models of osteoporosis. In addition, EVs isolated from stem cells have been considered for their regenerative capabilities. In cardiac applications, human iPSC-derived EVs enriched with cardiomyogenic miRNAs like miR-1 and miR-199a were generated by transducing iPSCs with lentiviral vectors containing these specific miRNAs [197]. These EVs improved cardiac repair by delivering miRNAs to cardiac fibroblasts, decreasing fibrosis and apoptosis, and increasing cardiomyocyte proliferation and cardiac function after MI. The engineered EVs not only serve to transport therapeutic miRNAs but also control the expression of genes associated with cardiogenesis and fibrosis, which makes these EVs multifunctional for cardiac regeneration.

#### Disease diagnosis

In the field of disease diagnosis, target-engineered EVs have been shown to hold potential as noninvasive biomarkers and imaging probes. The molecular content of EVs depends on their cellular source, and this has made them useful in the identification of different diseases. EVs have been investigated as biomarkers for early diagnosis of neurodegenerative diseases in which changes in the CNS can be identified through a non-invasive procedure. Winston *et al*. showed that tau pathology could be transmitted *in vivo* by engineered EVs produced from neuronally differentiated human-induced PSCs (NiPSCEs) [198]. The experimental design was to infect neurons differentiated from iPSC with tau-RD-LM-YFP adenovirus vectors to express the P301L and V337M mutations in the tau repeat domain. An immunohistochemical study confirmed that it was possible to transfer tau into the recipient mouse neurons by these exosomes, and this led to the development of widespread tau pathology. The pathology of tau inclusions was seen in several areas of the brain such as the hippocampus, thalamus, and cortex. The results of this study emphasize the possibilities of using engineered EVs in the investigation of the disease processes and the spread of pathology in neurodegenerative disorders such as Alzheimer’s disease and as tools for treatment and diagnosis.

Moreover, the engineered EVs can be armed with imaging agents for enhanced imaging whereby the disease processes can be seen in real time. This integration presents a new dimension in medical research. To this end, Engineered EVs provide enhanced imaging because they can become contrast agents that immediately and accurately depict pathological conditions. A very interesting area of application of engineered EVs in this regard is the possibility of transforming high-end imaging modalities, including magnetic resonance imaging (MRI), computed tomography, and single photon emission computed tomography [199]. Han and coworkers also proved that stem cell–derived EVs can be magnetically labeled with superparamagnetic iron oxide (SPIO) nanoparticles, and this enables efficient MRI monitoring of the EVs’ migration to the injured area [200]. Thus, SPIO nanoparticles with polyhistidine tags (SPIO-His) are encapsulated into EVs by electroporation. The magneto-EVs are then purified through the Ni-NTA affinity column to ensure high labeling efficiency and MRI sensitivity for *in vivo* tracking. This method increases the specificity of MRI and allows to control the efficiency of the therapeutic EV delivery accurately. Moreover, Liu *et al*. proved that the engineered EVs could be loaded with SPIO by the sonication process [201]. This procedure involves a short treatment of EVs with SPIO nanoparticles under ultrasonic conditions to achieve proper integration without disturbing the size or structure of the EVs. The SPIO labeling of EVs resulted in high MRI contrast and allowed for the accurate monitoring and imaging of the EVs in culture. They therefore hold a lot of promise for raising the standards of diagnosis and treatment in regenerative medicine.

#### Hydrogel delivery

Hydrogel-encapsulated EVs have been recently proposed as a promising strategy for targeted and prolonged drug delivery in regenerative medicine [202]. New approaches have been developed to create different kinds of hydrogels in order to ensure the appropriate stability and controlled release of iPSC-EVs. A collagen-based hydrogel patch was formulated to release iCM-EVs where iCMs were derived from iPSCs [203]. The use of collagen hydrogel was preferred because of its biocompatibility and capacity to give a time release of the material. Around 3 × 10^10^ iCM-EVs were loaded into the patch, and the vesicles were released at a slow rate over 21 days. This extended release was equally established through both *in vitro* and *in vivo* studies whereby the hydrogel was found to retain the EV bioactivity and thus offered continued therapeutic outcomes. There were improvements in the cardiac performance following myocardial infarction such as increased myocardial replacement, decreased size of the infarcted area, and increased contractility of the heart muscles. Likewise, an injectable photocurable Janus hyaluronic acid–*g*-(2-aminoethyl methacrylate hydrochloride–dopamine (HAD) hydrogel encapsulated iCM-Exos that have been fluorescently labeled with PKH26 [126]. The hydrogel had catechol groups to improve the attachment and maintain the release of exosomes for a longer time. Due to the photocuring ability of the hydrogel, it was possible to inject this hydrogel and cure it in the site of action, thus achieving the controlled release of EVs for 15 days and, consequently, the alleviation of oxidative stress and inflammation in myocardial infarction models. This method provided a high rate of retention and therapeutic effectiveness in a rat model of the cardiac postsurgery model with less pericardial adhesion, less oxidative stress, and less inflammatory response. In the case of corneal regeneration, a thermosensitive hydrogel was designed to deliver MSC-derived exosomes for the restoration of the corneal epithelium as well as stroma [195]. This hydrogel offered a time-dependent release of exosomes with encapsulated miRNAs for the treatment of tissue injury and scar minimization. The thermosensitive nature of the hydrogel made it to be in liquid form at low temperature and gel-like at body temperature, thus allowing for controlled and long-term release of the exosomes in the corneal tissue. This method revealed potential in a rat model as it revealed fewer scars, faster healing, less collagen deposition, and enhanced epithelium and stroma regeneration. Also, a microfiber-reinforced gelatin methacrylate (GelMA) hydrogel was designed for mechanical reinforcement and regulated EV release [204]. This system contained EVs that were obtained from hiPSC-EVs. Microfibers were used for the reinforcement of the hydrogel for the purpose of providing structural support to the hydrogel, which, in turn, released bioactive EVs in a controlled manner. It tried to control the release of EV for 14 days to enhance the migration of endothelial cells and the bioactivity of the materials, which was essential to stimulate the required cellular events for tissue repair. These studies taken together show that hydrogel-encapsulated EVs have the ability to provide long-term therapeutic delivery thus holding great promise in tissue repair and regeneration in many clinical applications. Here is the summary of these studies in Table 10.

**Table 10:** Applications of engineered vesicles derived from induced pluripotent stem cells and their derivative cells in various animal models

**Disease targeted**	**Vesicle source**	**Vesicle size** **(average diameter)**	**Engineered vesicle method**	**Animal model**	**Treatment outcome**	**Reference**
Heart	hiPSCs	EVs: 248.2 ± 107.2 nm; Magnetic EVs: 292.8 ± 113.0 nm	Magnetic EVs (1.1 × 10^11^ particles/ml) labeled with superparamagnetic iron oxide (SPIO) nanoparticles coated with polyhistidine tags (2 mg/ml) via electroporation	Mouse myocardial infarction	Significant protection and accumulation at injury	Han *et al*. [200]
Heart	hiPSC- cardiomyocytes (iCM)	EVs: 70–100 nm	A hydrogel patch composed of a 7 mm diameter collagen gel-foam mesh sustainably released encapsulated iCM-EVs (3 × 10^10^)	Rat myocardial infarction	Reduced arrhythmic burden, promoted ejection-fraction recovery, decreased cardiomyocyte apoptosis, reduced infarct size, and reduced cell hypertrophy	Liu *et al*. [203]
Heart	hiPSC- cardiomyocytes	Exosomes: 91 nm	Exosomes (0.1 μg/μl) encapsulated in injectable, photocurable Janus HAD hydrogel (3%)	Rat cardiac postsurgery model	Reduced pericardial adhesions, mitigated oxidative stress and inflammatory response	Wang *et al*. [126]
Heart	Genetically engineered hiPSCs and umbilical cord-MSCs	EVs: 110–120 nm	miR-1 and miR-199a enrichment via lentiviral transduction	*in vitro* study with human cardiac fibroblasts	Reduced apoptosis, decreased pro-inflammatory cytokines, downregulated pro-fibrotic gene α-SMA, induced cardiomyogenic gene expression	Katarzyna *et al*. [197]
Kidney	hiPSCs	EVs: 248.2 ± 107.2 nm; Magnetic EVs: 292.8 ± 113.0 nm	Magnetic EVs (1.1 × 10^11^ particles/ml) labeled with SPIO nanoparticles coated with polyhistidine tags (2 mg/ml) via electroporation	Mouse LPS-acute kidney injury	Selective accumulation at injury	Han *et al*. [200]
Brain	hiPSC-derived monocytes	EVs: 150.8 ± 19.6 nm	EVs were transfected with antisense miR-155–5p (αmiR) labeled with Cy5 and target monocytes in HIV-infected BLT mice	HIV-infected humanized bone marrow-liver-thymic (BLT) mouse model	Decreased T cell activation, altered monocyte activation markers, no decrease in viral load	Sun *et al*. [196]
Nerve	Neuronally-differentiated, human-induced pluripotent stem cells (NiPSCEs)	Exosomes: 100 nm	hiPSC- neurons overexpressing tau with P301L and V337M mutations by transfection with tau-RD-LM-YFP (P301L and V337M mutations)	Mouse Alzheimer’s disease (AD)	Human tau derived from neuronal exosomes is toxic to mouse neurons *in vivo*	Winston *et al*. [198]
Nerve	hiPSC- forebrain neural progenitor cortical organoids	EVs: 157.0 nm before sonication, 161.7 nm after sonication	EVs (1 x 10^11^ particles/ml) combined with SPIO (0.5 mg/ml) for sonication labeling	N/A	Enhanced MRI contrast for labeled EVs, feasible for *in vitro* tracking	Liu *et al*. [201]
Bone	hiPSC-MSCs	Bone-targeting (BT)-Exo (108 nm) or BT-Exo-siShn3 (118 nm)	BT-Exo (1 × 10^12^ particles/ml) loaded with 100 μg Shn3 siRNA via electroporation	Mouse osteoporosis	Enhanced bone regeneration and reduced osteoporosis symptoms​	Cui *et al*. [194]
Eye	hiPSC-MSCs	Exosomes: 70 nm	Exosomes (20 μg/ml) were loaded into thermosensitive chitosan-based hydrogel combined with glycerol 2-phosphate disodium salt hydrate (GP)	Rat corneal injury	Reduced scar formation, accelerated healing, downregulated collagen expression, and promoted epithelium and stroma regeneration	Tang *et al*. [195]
Vessel	hiPSCs	EVs: 50–200 nm	EVs (1.5 × 10^10^ particles/ml) encapsulated in microfiber-reinforced GelMA hydrogels (12%) with or without melt electrowritten reinforcing mesh	*in vitro* studies with human umbilical vein endothelial cells (HUVECs)	Prolonged EV release, increased endothelial migration, and enhanced bioactivity over 14 days	Cedillo-Servin *et al*. [204]

N/A means “not applicable”, only in vitro experiments, no animal research included

### Challenges and perspective

EV engineering is promising, but there are still technical challenges to be faced to fully meet this potential [151,152,205]. These challenges span across all engineering strategies, from genetic modifications to fusion strategies, and impact both the scalability and clinical applicability of EV-based therapies.

#### Technical challenges

(1) ‘Genetic engineering’ faces several challenges such as heterogeneity of parental cells, off-target effects, and scalability. The variability in donor cell populations leads to inconsistencies in EV size, cargo, and surface markers. Expression of specific proteins like tetraspanins at too high a level may disrupt other cellular processes. Virally transducing genetic modifications are laborious, costly, and hard to scale.

(2) ‘Surface modification’ faces several challenges such as stability of surface modifications, nonspecific binding, and efficiency of functionalization. *In vivo*, conjugated ligands or molecules may degrade or lose functionality because of enzymatic activity or harsh biological conditions. Modification of the cell surfaces may inadvertently cause nontarget cell interactions with the desired surface, reducing targeting efficiency. Existing techniques such as PEGylation or click chemistry find it difficult to produce uniform, reproducible modifications.

(3) ‘Payload loading techniques’ face several challenges such as low loading efficiency, cargo stability, and scalability of loading methods. The encapsulation efficiencies for passive loading are typically poor, while techniques such as electroporation damage EV membranes. During preparation and circulation, nucleic acids or proteins loaded into EVs may degrade. Sonication or freeze–thaw cycles are hard to scale for large-scale therapeutic applications.

(4) ‘Fusion strategies’ face several challenges such as complexity of hybrid systems, batch-to-batch variability, and safety concerns. Despite the fact that EVs are often combined with synthetic materials or liposomes, this always requires multistep processes, complicating production. However, the experimental conditions and starting materials differ, so attaining consistent fusion products remains challenging. *In vivo*, synthetic components may be immunogenic or toxic.

#### Future directions for optimization

(1) ‘Advanced biomanufacturing techniques’: Design and building of optimized bioreactors for large-scale manufacturing of engineered EVs with repeatability in size, composition, and purity. Genetic engineering and surface modification were automated and standardized reducing variability and improving reproducibility.

(2) ‘Improved targeting and loading efficiency’: The artificial intelligence–driven tools can be used to predict optimal cargo and surface modification strategies for target cell types and disease models. Novel active loading techniques for EVs, such as nanopores or microfluidic-based encapsulation, are explored toward increasing efficiency while preserving EV integrity.

(3) ‘Stability enhancements’: Additionally, the implementation of biomimetic coatings such as cell membrane cloaking to enhance EV stability and immune evasion may be investigated. Next-generation stabilizing agents or cross-linking strategies for the protection of EV cargo during systemic circulation may be developed.

(4) ‘Hybrid vesicle innovations’: Fusion protocols optimization to minimize production steps and reduce variability. Nanotechnology for use in integrated image, theranostic, and combination therapies.

#### Clinical translation potential

(1) ‘Regulatory challenges’: Creating regulatory standards that ensure effective, pure, and safe engineered EVs. EV quality, stability, and targeting efficiency are defined with standardized assays.

(2) ‘Preclinical and clinical validation’: The major aim is to perform large-scale preclinical studies determining the biodistribution, toxicity, and therapeutic efficacy of engineered EVs. Making humanized animal models for better prediction of clinical outcomes. Application to refining parameters such as dosage, routes of administration, and therapeutic distribution for clinical applications.

(3) ‘Cost and accessibility’: Improving scalability, therefore reducing high production costs through manufacturing processes streamlining. Affordable and accessible to wide clinical use.

(4) ‘Expanded applications’: In the future, it aims to study engineered EVs for emerging fields of immunotherapy, gene editing, and tissue engineering. Trying to find combination therapies to enhance current therapies like chemotherapy or biologics with EVs.

## Conclusions

Thus, the advancements described in this article illustrate that engineered EVs from PSCs can pave the way for regenerative medicine and numerous other applications. Due to their versatile design, such EVs, intended to provide individual cargo concerning the potency of stem cells and their capacity to repair numerous tissues, might have a high potential for addressing numerous therapeutic questions. With the engineered EVs, it is now possible to manipulate cellular behaviors, tissue regeneration, and potentially new therapeutic paradigms for numerous disease states in a targeted manner. These are renewable and are highly compatible with the host’s tissues and cells; they also elicit low immune responses, thereby making PSC–derived engineered EVs to be key players in the advancement of personalized and precision medicine. The biological role of EVs is better understood and with the developments in the creation of engineering EVs, the future lies in their use in clinical practice as safe and effective personalized therapies based on the use of PSCs for the treatment of patients in need across the world.
